# Investigations of sharp bounds for causal effects under selection bias

**DOI:** 10.1177/09622802251374168

**Published:** 2025-09-30

**Authors:** Stina Zetterstrom, Arvid Sjölander, Ingeborg Waernbaum

**Affiliations:** 1 Department of Statistics, 8097 Uppsala University, Uppsala, Sweden; 2 Department of Medical Epidemiology and Biostatistics, Karolinska Institute, Stockholm, Sweden

**Keywords:** Causal inference, inclusion criteria, risk difference, risk ratio, sensitivity analysis

## Abstract

Selection bias is a common type of bias, and depending on the causal estimand of interest and the structure of the selection variable, it can be a threat to both external and internal validity. One way to quantify the maximum magnitude of potential selection bias is to calculate bounds for the causal estimand. Here, we consider previously proposed bounds for selection bias, which require the specification of certain sensitivity parameters. First, we show that the sensitivity parameters are variation independent. Second, we show that the bounds are sharp under certain conditions. Furthermore, we derive improved bounds that are based on the same sensitivity parameters. Depending on the causal estimand, these bounds require additional information regarding the selection probabilities. We illustrate the improved bounds in an empirical example where the effect of breakfast eating on overweight is estimated. Lastly, the performance of the bounds are investigated in a numerical experiment for sharp and non-sharp cases.

## 1. Introduction

In observational studies, there are several sources of potential biases when estimating a causal effect of an exposure on an outcome of interest. One type of bias in observational studies is selection bias which can occur when the study is conducted in a subset of a population. Intuitively, selection bias can arise when the target population is the total population, that is, one wishes to generalize the results to subjects in both the selected and non-selected part of the population. However, selection bias can also arise when the target population is the selected part of the population. Commonly, when no inclusion criteria are employed, the estimand of interest lies in the total population, and when inclusion criteria are used, the interest instead often lies in the subpopulation estimand. To assess the maximum magnitude of the potential selection bias, a sensitivity analysis can be used, e.g. calculating bounds for the causal estimands under selection bias. Several analytical bounds have been proposed, both for total population estimands and subpopulation estimands.^1–5^ Alternatively, Duarte et al.^6^ propose an algorithm for deriving numerical bounds.

Here, we build upon Smith and VanderWeele,^7^ hereafter referred to as SV. These authors developed bounds that require the analyst to specify certain sensitivity parameters under specific conditional independence assumptions. The sensitivity parameters describe the maximum strength of the dependence between the selection variable, the outcome, the exposure and an unmeasured variable. However, SV did not discuss whether the sensitivity parameters are *variation independent* of each other and the observed data distribution. This is a desirable property since the sensitivity parameters can be specified individually without taking the value of the other sensitivity parameters into account. Furthermore, SV did not discuss whether their bounds are *sharp* relative to the necessary assumption and information when the sensitivity parameters and data distribution fulfill specific criteria. In this work, we investigate the SV bounds in similar ways as Sjölander^8^ did for bounds for causal estimands under confounding. More specifically, we derive the feasible regions of the sensitivity parameters and show that they are variation independent of each other and the observed data distribution, and we show that the SV bounds are sharp under specific criteria, that is, they are the tightest possible bounds under the necessary assumptions, and that they are non-sharp when the criteria are not fulfilled, that is, tighter bounds can be found. Furthermore, we propose improved sharp bounds using the same sensitivity parameters as the SV bounds, noting that they require additional knowledge of the data in some instances. The improved bounds coincide with SV’s bounds in certain areas and are tighter in others. The bounds and how they can be used are illustrated in an empirical example investigating the causal effect of breakfast-eating on overweight. The performance of the bounds in comparison to the SV bounds are evaluated in a numerical example. Here, we show that the improved bounds are typically tighter when the additional knowledge is used, but that the two bounds are similar when the additional knowledge is not used.

The rest of the article is structured as follows. In Section 2, we present notation, definitions, and assumptions and briefly report SV’s bounds. In Section 3, we derive the theoretical properties of the SV bounds and present the improved bounds in Section 4. We illustrate the improved bounds in an empirical example and numerical example in Sections 5 and 6, and finally, discuss the results in Section 7.

## 2. Theoretical framework

### 2.1. Notations, definitions, and assumptions

Notation is presented in throughout in the text, and a summary is found in Supplemental Appendix A. We use the Neyman-Rubin causal model^9,10^ to define potential outcomes, $Y^{a}$ , for each subject, had that subject been exposed to exposure A=a . Furthermore, let A , Y , and S be the exposure, outcome, and selection indicator, respectively, all assumed to be binary. The selection indicator variable defines an infinitely large subpopulation (i.e. the data generating mechanism) from which a particular study sample is taken, not inclusion in the particular study sample per se. We assume that the potential outcome is related to the observed outcome as

(1) $$Y=AY^{1}+(1-A)Y^{0}$$

which is usually referred to as consistency. Throughout, we assume that the analysis is performed conditional on a set of pre-exposure covariates, X=x , that is sufficient for confounding control; however, to keep notation simple we keep the conditioning on X implicit in all expressions. We define the risk difference and risk ratio in the selected subpopulation as ${\text{RD}}_{S}=p(Y=1\;|\;A=1,S=1)-p(Y=1\;|\;A=0,S=1)$ and ${\text{RR}}_{S}=p(Y=1\;|\;A=1,S=1)/p(Y=1\;|\;A=0,S=1)$ . To ease the notation, we use p(⋅) to denote both probabilities and distributions. The risk difference or risk ratio is estimated from data. However, we assume that there is no sampling variability and that ${\text{RD}}_{S}$ and ${\text{RR}}_{S}$ are population quantities.

If the interest lies in the total population, that is, the target is to generalize the results to subjects in both the selected and non-selected part of the population, the target estimand is $p(Y^{a}=1)$ , for a∈{0,1} , or some contrast thereof, for example, the causal risk difference ${\text{CRD}}_{T}=p(Y^{1}=1)-p(Y^{0}=1)$ or the causal risk ratio ${\text{CRR}}_{T}=p(Y^{1}=1)/p(Y^{0}=1)$ . To make inference on such total population estimands, we follow SV and assume the existence of an unobserved (set of) variable(s) U such that

(2) Ya⊥⊥A|U,11a∈{0,1}

and

(3) Y⊥⊥S|(A,U)

Under (1) to (3), we can rewrite $p(Y^{a}=y)=E_{U}(p(Y=y\;|\;S=1,A=a,U))$ , see details in Supplemental Appendix B. In order to take the average $E_{U}(p(Y=y\;|\;S=1,A=a,U))$ , we have to require that p(Y=y|A=a,S=1,U) is well defined. Assumption (3) is strong in many real-world applications and careful utilization is recommended.

On the other hand, if the specific subpopulation is of interest, that is, the target is to generalize the results only to subjects in the selected part of the population, the target estimand is instead $p(Y^{a}=1\;|\;S=1)$ , for a∈{0,1} or some contrast thereof, for example, the causal risk difference ${\text{CRD}}_{S}=p(Y^{1}\;|\;S=1)-p(Y^{0}\;|\;S=1)$ or the causal risk ratio ${\text{CRR}}_{S}=p(Y^{1}\;|\;S=1)/p(Y^{0}\;|\;S=1)$ . To make inference on such subpopulation estimands, we follow SV and assume the existence of an unobserved (set of) variable(s) U such that

(4) $$Y^{a}⊥⊥A\;|\;(S=1,U)$$

Under (1) and (4), we can rewrite $p(Y^{a}=y\;|\;S=1)=E_{U}(p(Y=y\;|\;S=1,A=a,U)\;|\;S=1)$ , see details in Supplemental Appendix B. Again, in order to take the average $E_{U}(p(Y=y\;|\;S=1,A=a,U)\;|\;S=1)$ , we have to require that p(Y=y|A=a,S=1,U) is well defined.

We caution the reader that Assumption (4) for the selected subpopulation is more subtle than the corresponding Assumption (2) for the total population, and that there are realistic situations where, for a given set of variables U , the independence in (4) is violated even though the independence in (2) is not. We give one such example in Supplemental Appendix C, for which SV incorrectly assumed the independence in (4) to hold.

In this work, selection bias is measured on the same scale as the estimand, so for the risk ratios the bias is defined as $B_{{\mathrm{RR}}_{T}}={\mathrm{RR}}_{S}/{\mathrm{CRR}}_{T}$ , and for the risk difference as $B_{{\mathrm{RD}}_{T}}={\mathrm{RD}}_{S}-{\mathrm{CRD}}_{T}$ . Here, we add to the literature on bounds for causal estimands under selection bias. In other words, the bounds constructed here fulfill ${\mathrm{LB}}_{\mathrm{CE}}\leq {\mathrm{CRR}}_{T}\leq {\mathrm{UB}}_{\mathrm{CE}}$ , where ${\mathrm{LB}}_{\mathrm{CE}}$ and ${\mathrm{UB}}_{\mathrm{CE}}$ are the lower and upper bounds of the causal estimand. Bounds can also be derived directly for the bias^7^ (e.g. ${\mathrm{LB}}_{B}\leq B\leq {\mathrm{UB}}_{B}$ ). The bounds for causal estimands under the selection bias are readily transformed to bounds for the bias as ${\mathrm{RR}}_{S}/{\mathrm{UB}}_{\mathrm{CE}}\leq B\leq {\mathrm{RR}}_{S}/{\mathrm{LB}}_{\mathrm{CE}}$ (analogously for the risk difference). The same definitions apply for ${\mathrm{CRR}}_{S}$ and ${\mathrm{CRD}}_{S}$ .

### 2.2. Illustration using the NHANES data example

**Figure 1.**
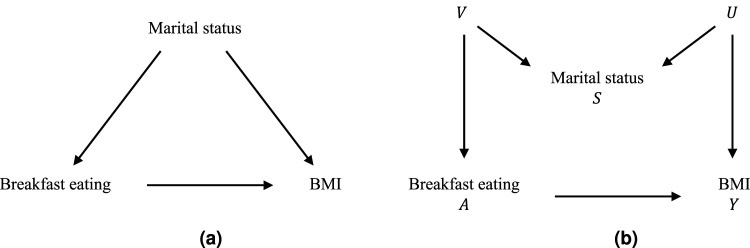
Possible structures for the selection where (a) marital status is a confounder and (b) a collider.

The effect of breakfast-eating on body mass index (BMI) has previously been studied using the National Health and Nutrition Examination Survey (NHANES) data, 1999-2000, and Song et al.^11^ studied whether skipping breakfast is associated with BMI in US adults. Here, we use this example to illustrate the assumptions and bounds using NHANES data from 1999 to 2018.^12^ The original article includes several covariates in the analysis, but we focus on one stratum in line with the setting of this article. The stratum considered is reliable responders, men, 30–39 years old, non-hispanic white, non-smokers, and non-exercisers. Furthermore, marital status can be considered to be a proxy variable for other variables that may be correlated with breakfast eating and/or overweight and are more difficult to quantify, for example, socioeconomic status and lifestyle habits. If a selection on marital status is made, and only subjects who are married/living with a partner are included, one might think that the bias is reduced (Figure 1(a)) when instead marital status is not a confounder, and the bias is increased due to selection bias from a potential M-structure (Figure 1(b)). Here, the unknown variable U can for example be education. If this type of variable exist, then Assumption (3) holds, and marital status is independent of BMI, conditional on breakfast-eating and education. Furthermore, Assumption (4) is also fulfilled, and the potential outcomes are independent of breakfast-eating among the married/co-habitants, conditional on education.

### 2.3. Sensitivity parameters in SV’s bounds

The sensitivity parameters in the SV bounds are constructed as risk ratios that describe the maximum strengths of dependencies between the unmeasured variable *U* in relations (2) to (4), and the other variables. For the total population, SV defined the sensitivity parameters as follows:

$$\begin{array}{rl} {\text{RR}}_{SU|as} & =\max_{u}\frac{p(U=u\;|\;S=s,A=a)}{p(U=u\;|\;S=1-s,A=a)} \end{array}$$

$$\begin{array}{rl} {\text{RR}}_{UY|a} & =\frac{\max_{u}p(Y=1\;|\;U=u,A=a)}{\min_{u}p(Y=1\;|\;U=u,A=a)} \end{array}$$

and

$$\begin{array}{r} {\text{BF}}_{as}=\frac{{\text{RR}}_{SU|as}\times {\text{RR}}_{UY|a}}{{\text{RR}}_{SU|as}+{\text{RR}}_{UY|a}-1} \end{array}$$

SV derived lower bounds for the CRR in the total population,

$$\begin{array}{r} {\text{CRR}}_{T}\geq \frac{{\text{RR}}_{S}}{{\text{BF}}_{11}{\text{BF}}_{00}} \end{array}$$

and the CRD in the total population,

$$\begin{array}{r} {\text{CRD}}_{T}\geq p(Y=1\;|\;A=1,S=1)\left(1+\frac{1}{{\mathrm{BF}}_{11}}\right)-p(Y=1\;|\;A=0,S=1)(1+{\mathrm{BF}}_{00})-{\mathrm{BF}}_{11} \end{array}$$

For the subpopulation, SV defined the sensitivity parameters as follows:

$$\begin{array}{rl} {\text{RR}}_{AU|a} & =\max_{u}\frac{p(U=u\;|\;S=1,A=a)}{p(U=u\;|\;S=1,A=1-a)} \end{array}$$

$$\begin{array}{rl} {\text{RR}}_{UY|S=1} & =\max_{a}\frac{\max_{u}p(Y=1\;|\;U=u,A=a,S=1)}{\min_{u}p(Y=1\;|\;U=u,A=a,S=1)} \end{array}$$

and

$$\begin{array}{r} {\text{BF}}_{a}=\frac{{\text{RR}}_{AU|a}\times {\text{RR}}_{UY|S=1}}{{\text{RR}}_{AU|a}+{\text{RR}}_{UY|S=1}-1} \end{array}$$

From these sensitivity parameters, SV constructed lower bounds for the CRR in the subpopulation,

$$\begin{array}{r} {\text{CRR}}_{S}\geq {\text{RR}}_{S}/{\text{BF}}_{1} \end{array}$$

and the CRD in the subpopulation,

$$\begin{array}{r} {\text{CRD}}_{S}\geq {\text{RR}}_{S}-\max[p(Y=1\;|\;A=0,S=1)({\mathrm{BF}}_{1}-1),p(Y=1\;|\;A=1,S=1)(1-1/{\mathrm{BF}}_{1})] \end{array}$$

Note that p(U=u|A=a,S=s)>0 for all values of a and s in order to avoid division by 0.

The sensitivity parameters measures the potential magnitude of the maximum selection bias. If no selection bias is present, they are equal to 1. Interpretations of the sensitivity parameters have previously been discussed.^5^ In terms of the NHANES example, the sensitivity parameters ${\text{RR}}_{SU|as}$ can be interpreted as the maximum selection ratio among each group of breakfast eaters and ${\text{RR}}_{UY|a}$ can be thought of as the maximum risk ratio of education on BMI among each group of the breakfast eaters. For the subpopulation, ${\text{RR}}_{AU|a}$ can be interpreted as maximum risk ratio of breakfast-eating on education for the married/co-habitants and ${\text{RR}}_{UY|S=1}$ is the maximum risk ratio of education on BMI for either those who eat or skip breakfast. It may be difficult to specify these sensitivity parameters. The assumed values can, for example, be based on expert knowledge, or previous studies. The sensitivity parameters can also be calculated for a measured variable which can give a plausible range of values to consider.

SV only presented lower bounds, both in the total and subpopulation, and they suggested that the exposure variable should be recoded when the upper bound is of interest. To simplify for the data-analysts, we have constructed an upper bound that is equal to the lower bound with the recoded exposure.

## 3. Properties of SV’s bounds

### 3.1. Feasible regions

It is desirable to set sensitivity parameters to values that are logically possible based on their definitions. Thus, we start with deriving the sets of logically possible values for the sensitivity parameters, that is, their feasible regions. Sensitivity parameters can be restricted by, for example, the data, their definitions or by each other. If a sensitivity parameter is not restricted by another quantity, then it is said to be variation independent of that quantity. Variation independence is desirable because it simplifies for user as the sensitivity parameters can be considered separately. Theorem 1 considers feasible regions and variation independence for the sensitivity parameters for the total population:

Theorem 1
$\{{\text{RR}}_{SU|00},$ ${\text{RR}}_{SU|01},$ ${\text{RR}}_{SU|10},$ ${\text{RR}}_{SU|11},$ ${\text{RR}}_{UY|0},$ ${\text{RR}}_{UY|1}\}$ are restricted by their definitions to be equal to or greater than 1. Furthermore, {p(Y,A|S=1), p(S=1|A=1), p(S=1|A=0), ${\text{RR}}_{SU|00},$ ${\text{RR}}_{SU|01},$ ${\text{RR}}_{SU|10},$ ${\text{RR}}_{SU|11},$ ${\text{RR}}_{UY|0},$ ${\text{RR}}_{UY|1}\}$ form a variationally independent parametrization of a joint distribution p(Y,A,S,U) encoding Assumption (3) for binary Y,A,S .

The reason for caring about variation independence of p(S=1|A=1) and p(S=1|A=0) is that we use these in the construction of bounds below. Theorem 2 considers feasible regions and variation independence for the sensitivity parameters for the subpopulation:

Theorem 2
$\{{\text{RR}}_{UY|S=1}$ , ${\text{RR}}_{AU|1}$ , ${\text{RR}}_{AU|0}\}$ are restricted by their definitions to values equal to or greater than 1. Furthermore, $\{{\text{RR}}_{UY|S=1}$ , ${\text{RR}}_{AU|1}$ , ${\text{RR}}_{AU|0}$ , p(Y,A|S=1)} form a variationally independent parametrization of a joint distribution p(Y,A,U|S=1) encoding Assumption (4) for binary Y,A,S .

Theorems 1 and 2 imply that the users of the bounds can consider all values >1 as logically possible, although they might not be equally plausible. The proofs of Theorems 1 and 2 are given in Supplemental Appendices D and E. See Chen,^13^ Nabi et al.,^14^ Malinsky et al.,^15^ Shpitser^16^ for results on variation independent parameters in other settings. Note that it is enough to show variation independence for one specific distribution because that means that there exists (at least) one unmeasured variable U such that variation independence holds, and since U is unmeasured, it could possibly be this variable. This does not mean that it necessarily is that variable. The same argument is used in the proofs for sharpness.

### 3.2. Results of sharpness for SV bounds

A bound is valid if it contains the true causal estimand. Furthermore, a bound is sharp if the bias can be equal to the value of the bound, for an observed distribution and correctly specified sensitivity parameters. Thus, a sharp bound is the tightest valid bound. Bounds are derived under specific assumptions and information, and a bound is thus sharp under its necessary assumptions and information. Thus, two different bounds for the same causal estimand can be simultaneously sharp if they are derived under different assumptions and information. We emphasize that a bound can be valid even though it is not sharp.

The SV bounds for the risk ratio in the total population are sometimes arbitrarily sharp, in the sense that the selection bias can be arbitrarily close the bound, but not exactly equal. More details are given in Supplemental Appendix F. In Theorem 3, we present sufficient conditions for when the SV bounds for the risk ratio in the total population are arbitrarily sharp. Theorem 3 is proved in Supplemental Appendix F.

Theorem 3 Given Assumptions (1)–(3) and $\{{\text{RR}}_{SU|00},$ ${\text{RR}}_{SU|11},$ ${\text{RR}}_{UY|0},$ ${\text{RR}}_{UY|1}\}$ and p(Y,A,U|S=1) are such that ${\text{BF}}_{00}\leq 1/p(Y=1\;|\;A=0,S=1)$ and that $p(S=1\;|\;A=a)<\delta_{a}$ , where $0<\delta_{a}<1$ for both a∈{0,1} , then is the lower bound for ${\text{CRR}}_{T}$ arbitrarily sharp.

Given Assumptions (1)–(3) and $\{{\text{RR}}_{SU|01},$ ${\text{RR}}_{SU|10},$ ${\text{RR}}_{UY|0},$ ${\text{RR}}_{UY|1}\}$ and p(Y,A,U|S=1) are such that ${\text{BF}}_{10}\leq 1/p(Y=1\;|\;A=1,S=1)$ and that $p(S=1\;|\;A=a)<\delta_{a}$ , where $0<\delta_{a}<1$ for both a∈{0,1} , then is the upper bound for ${\text{CRR}}_{T}$ arbitrarily sharp.

Thus, if ${\text{BF}}_{as}>1/p(Y=1\;|\;A=a,S=s)$ or if $p(S=1\;|\;A=a)>\delta_{a}$ for both a∈{0,1} , the bounds are valid but the bias cannot be as large as the bounds suggests, that is, the bounds are too conservative.

The lower SV bound for the risk difference in the total population is generally not sharp, except for the very specific condition $p(Y=1\;|\;A=1,S=1)-p(Y=1\;|\;A=0,S=1)={\mathrm{BF}}_{11}$ . Similar arguments can be made for the upper SV bound. This will be further clarified in Section 4.

The SV bounds for the risk ratio in the subpopulation are sometimes sharp. In Theorem 4, which is proved in Supplemental Appendix G, we present a necessary and sufficient condition for when the SV bounds for the risk ratio in the subpopulation are sharp.

Theorem 4 Given Assumptions (1) and (4) and $\{{\text{RR}}_{UY|S=1}$ , ${\text{RR}}_{AU|1}$ , ${\text{RR}}_{AU|0}\}$ and p(Y,A,U|S=1) are such that ${\text{BF}}_{1}\leq 1/p(Y=1\;|\;A=0,S=1)$ , then and only then is the lower bound for ${\text{CRR}}_{S}$ sharp.

Given Assumptions (1) and (4) and $\{{\text{RR}}_{UY|S=1}$ , ${\text{RR}}_{AU|1}$ , ${\text{RR}}_{AU|0}\}$ and p(Y,A,U|S=1) are such that ${\text{BF}}_{0}\leq 1/p(Y=1\;|\;A=1,S=1)$ , then and only then is the upper bound for ${\text{CRR}}_{S}$ sharp.

Thus, if ${\text{BF}}_{a}>1/p(Y=1\;|\;A=1-a,S=1)$ , the bound is valid but the bias cannot be as large as the bounds suggests.

The SV bounds for the risk difference in the subpopulation are arbitrarily sharp. Sufficient and necessary conditions are presented in Theorem 5, proved in Supplemental Appendix H.

Theorem 5 Given Assumptions (1) and (4) and $\{{\text{RR}}_{UY|S=1}$ , ${\text{RR}}_{AU|1}$ , ${\text{RR}}_{AU|0}\}$ and p(Y,A,U|S=1) are such that ${\text{BF}}_{1}\leq 1/p(Y=1\;|\;A=0,S=1)$ and that $p(A=0\;|\;S=1)<\delta_{0}$ , where $0<\delta_{0}<1$ , if $p(Y=1\;|\;A=0,S=1)({\mathrm{BF}}_{1}-1)<p(Y=1\;|\;A=1,S=1)(1-1/{\mathrm{BF}}_{1})$ or that $p(A=1\;|\;S=1)<\delta_{1}$ , where $0<\delta_{1}<1$ , if $p(Y=1\;|\;A=0,S=1)({\mathrm{BF}}_{1}-1)>p(Y=1\;|\;A=1,S=1)(1-1/{\mathrm{BF}}_{1})$ , then and only then is the lower bound for ${\text{CRD}}_{S}$ arbitrarily sharp.

Given Assumptions (1) and (4) and $\{{\text{RR}}_{UY|S=1}$ , ${\text{RR}}_{AU|1}$ , ${\text{RR}}_{AU|0}\}$ and p(Y,A,U|S=1) are such that ${\text{BF}}_{0}\leq 1/p(Y=1\;|\;A=1,S=1)$ and that $p(A=0\;|\;S=1)<\delta_{0}$ , where $0<\delta_{0}<1$ , if $p(Y=1\;|\;A=0,S=1)({\mathrm{BF}}_{1}-1)<p(Y=1\;|\;A=1,S=1)(1-1/{\mathrm{BF}}_{1})$ or that $p(A=1\;|\;S=1)<\delta_{1}$ , where $0<\delta_{1}<1$ , if $p(Y=1\;|\;A=0,S=1)({\mathrm{BF}}_{1}-1)>p(Y=1\;|\;A=1,S=1)(1-1/{\mathrm{BF}}_{1})$ , then and only then is the upper bound for ${\text{CRD}}_{S}$ arbitrarily sharp.

Thus, if ${\text{BF}}_{a}>1/p(Y=1\;|\;A=1-a,S=1)$ or if $p(A=a\;|\;S=1)>\delta_{a}$ for a∈{0,1} , the bounds are valid but the bias cannot be as large as the bounds suggests. However, one can construct similar bounds that do not require $p(A=a\;|\;S=1)<\delta_{a}$ to be sharp by using results from Ding and VanderWeele.^17^ The bounds are

(5) $${\mathrm{RD}}_{\mathrm{obs}}-{\tilde{\mathrm{BF}}}_{1}\leq {\mathrm{CRD}}_{S}\leq {\mathrm{RD}}_{\mathrm{obs}}+{\tilde{\mathrm{BF}}}_{0}$$

where

$$\begin{array}{rl} {\tilde{\mathrm{BF}}}_{a} & =p(A=1-a\;|\;S=1)\cdot p(Y=1\;|\;A=a,S=1)\cdot (1-1/{\mathrm{BF}}_{a})\\ & \;+p(A=a\;|\;S=1)\cdot p(Y=1\;|\;A=1-a,S=1)\cdot ({\mathrm{BF}}_{a}-1). \end{array}$$

These bounds are sharp when ${\text{BF}}_{a}<1/p(Y=1\;|\;A=1-a,S=1)$ , that is, under the same conditions as the risk ratio in the subpopulation.

A consequence of the results on sharpness is that one can obtain improved bounds, that is, bounds that are equal to the SV bounds when they are sharp and tighter in the region where the SV bounds are not sharp. Such bounds are presented in the next section.

## 4. Improved bounds

### 4.1. Total population

The SV bounds in the total population are not sharp under certain conditions, as shown in the previous section, but the sensitivity parameters can be used to construct improved bounds that are generally sharp. Define

$$\begin{array}{r} l_{a}=p(Y=1\;|\;A=a,S=1)\{p(S=1\;|\;A=a)+p(S=0\;|\;A=a)/{\text{BF}}_{a1}\} \end{array}$$

and

$$\begin{array}{rl} u_{a} & =p(Y=1\;|\;A=a,S=1)\\ & \;\times \left[p(S=1\;|\;A=a)+p(S=0\;|\;A=a)\times \text{min}\{{\text{BF}}_{a0},1/p(Y=1\;|\;A=a,S=1)\}\right] \end{array}$$

and consider the following bounds for $p(Y^{a}=1)$ :

(6) $$l_{a}\leq p(Y^{a}=1)\leq u_{a}$$

Upper (lower) bounds for the CRR are found by combining $l_{0}$ ( $l_{1}$ ) and $u_{1}$ ( $u_{0}$ ). In Supplemental Appendix I, we show that the bounds in (6) have two important properties, which we summarize in a theorem.

Theorem 6

(a) The bounds $\left(l_{a},u_{a}\right)$ are valid, in the sense that the inequalities in (6) hold for all distributions p(Y,A,S,U) .
(b) The bounds $\left(l_{1},u_{0}\right)$ are simultaneously sharp, in the sense that, for any specific $\{p^{*}(Y,A\;|\;S=1),$ $p^{*}(S=1\;|\;A=1),$ $p^{*}(S=1\;|\;A=0),$ ${\text{RR}}_{SU|00}^{*},$ ${\text{RR}}_{SU|11}^{*},$ ${\text{RR}}_{UY|0}^{*},$ ${\text{RR}}_{UY|1}^{*}\}$ , there exists a distribution p(Y,A,S,U) for which Assumptions (1)–(3) holds, such that {p(Y,A|S=1), p(S=1|A=1), p(S=1|A=0), ${\text{RR}}_{SU|00},$ ${\text{RR}}_{SU|11},$ ${\text{RR}}_{UY|0},$ ${\text{RR}}_{UY|1}\}$ = $\{p^{*}(Y,A\;|\;S=1),$ $p^{*}(S=1\;|\;A=1),$ $p^{*}(S=1\;|\;A=0),$ ${\text{RR}}_{SU|00}^{*},$ ${\text{RR}}_{SU|11}^{*},$ ${\text{RR}}_{UY|0}^{*},$ ${\text{RR}}_{UY|1}^{*}\}$ , $p(Y=1\;|\;A=1)=l_{1}$ and $p(Y=1\;|\;A=0)=u_{0}$ .
(c) The bounds $\left(l_{0},u_{1}\right)$ are simultaneously sharp, in the sense that, for any specific $\{p^{*}(Y,A\;|\;S=1),$ $p^{*}(S=1\;|\;A=1),$ $p^{*}(S=1\;|\;A=0),$ ${\text{RR}}_{SU|01}^{*},$ ${\text{RR}}_{SU|10}^{*},$ ${\text{RR}}_{UY|0}^{*},$ ${\text{RR}}_{UY|1}^{*}\}$ , there exists a distribution p(Y,A,S,U) for which Assumptions (1)–(3) holds, such that {p(Y,A|S=1), p(S=1|A=1), p(S=1|A=0), ${\text{RR}}_{SU|01},$ ${\text{RR}}_{SU|10},$ ${\text{RR}}_{UY|0},$ ${\text{RR}}_{UY|1}\}$ = $\{p^{*}(Y,A\;|\;S=1),$ $p^{*}(S=1\;|\;A=1),$ $p^{*}(S=1\;|\;A=0),$ ${\text{RR}}_{SU|01}^{*},$ ${\text{RR}}_{SU|10}^{*},$ ${\text{RR}}_{UY|0}^{*},$ ${\text{RR}}_{UY|1}^{*}\}$ , $p(Y=1\;|\;A=1)=u_{1}$ and $p(Y=1\;|\;A=0)=l_{0}$ .

To be able to use these bounds in practice, one must know, or have a reasonable guess, the sampling proportion p(S=1|A=a) and the bias factors $\left({\text{BF}}_{a0},{\text{BF}}_{a1}\right)$ . If either of these is unknown, then one can obtain bounds that can be used by minimizing and maximizing the lower and upper bounds in (6), respectively, with respect to the unknown quantities. The lower bound and upper bounds in (6) are monotonically increasing and decreasing in p(S=1|A=a) , respectively, so they are minimized and maximized by setting p(S=1|A=a)=0 . We then obtain the bounds as follows:

(7) $$\begin{array}{rl} p(Y & =1\;|\;A=a,S=1)/{\text{BF}}_{a1}\\ & \leq p(Y=1\;|\;A=a)\leq \\ p(Y & =1\;|\;A=a,S=1)\times \text{min}\{{\text{BF}}_{a0},1/p(Y=1\;|\;A=a,S=1)\} \end{array}$$

which can be used if p(S=1|A=a) is unknown. The bounds in (7) give a lower bound for ${\mathrm{CRR}}_{T}$ that is identical to SV’s lower bound if ${\text{BF}}_{00}<1/p(Y=1\;|\;A=0,S=1)$ , but is otherwise tighter. Similarly to the SV bound, when p(S=1|A=a)→0 , the bound is sharp. From (7), it can be seen that the SV bound for the risk difference in the total population is not sharp unless very specific conditions are fulfilled. A lower bound for ${\mathrm{CRD}}_{T}$ is

$$\begin{array}{r} p(Y=1\;|\;A=a,S=1)\left(\frac{1}{{\mathrm{BF}}_{11}}\right)-p(Y=1\;|\;A=0,S=1){\mathrm{BF}}_{00} \end{array}$$

The lower SV bound is only equal to the above bound when $p(Y=1\;|\;A=1,S=1)-p(Y=1\;|\;A=0,S=1)={\mathrm{BF}}_{11}\geq 1$ , which most often will not be the case and the SV bound is thus not sharp.

The lower bound and upper bounds in (6) are monotonically decreasing and increasing in ${\text{BF}}_{a1}$ and ${\text{BF}}_{a0}$ , respectively, so they are minimized and maximized by setting ${\text{BF}}_{a1}={\text{BF}}_{a0}=\infty$ . We thus obtain the bounds

(8) $$\begin{array}{rl} p(Y & =1\;|\;A=a,S=1)p(S=1\;|\;A=a)\\ & \leq p(Y=1\;|\;A=a)\leq \\ p(Y & =1\;|\;A=a,S=1)p(S=1\;|\;A=a)+p(S=0\;|\;A=a) \end{array}$$

which can be used if $\left({\text{BF}}_{a0},{\text{BF}}_{a1}\right)$ are unknown.

### 4.2. Subpopulation

The SV bounds in the subpopulation are only sharp under specific conditions, as shown in the previous section, but the sensitivity parameters can be used to construct improved bounds that are generally sharp. We define

$$\begin{array}{r} l_{a}^{\prime }=p(Y=1\;|\;A=a,S=1)\{p(A=a\;|\;S=1)+p(A=1-a\;|\;S=1)/{\text{BF}}_{a}\} \end{array}$$

and

$$\begin{array}{rl} u_{a}^{\prime } & =p(Y=1\;|\;A=a,S=1)\times \left[p(A=a\;|\;S=1\right)\\ & \;+p(A=1-a\;|\;S=1)\times \text{min}\{{\text{BF}}_{\left(1-a\right)},1/p(Y=1\;|\;A=a,S=1)\}] \end{array}$$

and consider the following bounds for $p(Y^{a}=1\;|\;S=1)$ :

(9) $$l_{a}^{\prime }\leq p(Y^{a}=1\;|\;S=1)\leq u_{a}^{\prime }$$

In Supplemental Appendix J, we show that the bounds in (9) have two important properties, which we summarize in a theorem.

Theorem 7

(a) The bounds $\left(l_{a}^{\prime },u_{a}^{\prime }\right)$ are valid, in the sense that the inequalities in (9) hold for all distributions p(Y,A,U|S=1) .
(b) The bounds $\left(l_{1}^{\prime },u_{0}^{\prime }\right)$ are simultaneously sharp, in the sense that, for any specific $\{p^{*}(Y,A\;|\;S=1),$ ${\text{RR}}_{AU|1}^{*},$ ${\text{RR}}_{UY|S=1}^{*}\}$ , there exists a distribution p(Y,A,U|S=1) for which Assumptions (1) and (4) holds, such that {p(Y,A|S=1), ${\text{RR}}_{AU|1},$ ${\text{RR}}_{UY|S=1}\}$ = $\{p^{*}(Y,A\;|\;S=1),$ ${\text{RR}}_{AU|1}^{*},$ ${\text{RR}}_{UY|S=1}^{*}\}$ , $p(Y^{1}=1\;|\;S=1)=l_{1}^{\prime }$ and $p(Y^{0}=1\;|\;S=1)=u_{0}^{\prime }$ .
(c) The bounds $\left(l_{0}^{\prime },u_{1}^{\prime }\right)$ are simultaneously sharp, in the sense that, for any specific $\{p^{*}(Y,A\;|\;S=1),$ ${\text{RR}}_{AU|0}^{*},$ ${\text{RR}}_{UY|S=1}^{*},\}$ , there exists a distribution p(Y,A,U|S=1) for which Assumptions (1) and (4) holds, such that {p(Y,A|S=1), ${\text{RR}}_{AU|0},$ ${\text{RR}}_{UY|S=1}\}$ = $\{p^{*}(Y,A\;|\;S=1),$ ${\text{RR}}_{AU|0}^{*},$ ${\text{RR}}_{UY|S=1}^{*}\}$ , $p(Y^{1}=1\;|\;S=1)=u_{1}^{\prime }$ and $p(Y^{0}=1\;|\;S=1)=l_{0}^{\prime }$ .

A result of Theorem 7 is that one can construct sharp lower (upper) bounds for any contrast between $p(Y^{1}=1\;|\;S=1)$ and $p(Y^{0}=1\;|\;S=1)$ by contrasting $l_{1}^{\prime }$ ( $u_{1}^{\prime }$ ) and $l_{0}^{\prime }$ ( $u_{0}^{\prime }$ ). The improved bounds in (9) give bounds for ${\mathrm{CRR}}_{S}$ that coincide with the SV bounds in the regions where the SV bounds are sharp, that is, when ${\mathrm{BF}}_{a}\leq 1/p(Y=1\;|\;A=1-a,S=1)$ for a∈{0,1} . However, if ${\mathrm{BF}}_{a}>1/p(Y=1\;|\;A=1-a,S=1)$ , the improved bounds are tighter.

## 5. Empirical example

Here, we demonstrate the improved bounds by revisiting the NHANES example where the effect of breakfast-eating on overweight (BMI>25) using NHANES data from 1999 to 2018 is investigated.^12^ The original article includes several covariates in the analysis, but we analyze one stratum in line with the setting of this article. If more strata are of interest, the sensitivity analysis will have to be repeated in each stratum. There are 576 subjects in the chosen stratum where 436 subjects are selected (married or living with a partner). Among all subjects (selected and non-selected), 473 are breakfast eaters, and 103 are breakfast skippers. Among the selected subjects, 371 eat breakfast and 65 do not eat breakfast. Among the selected breakfast eaters, 239 subjects are overweight, and among the selected breakfast skippers, 53 are overweight. Thus, we obtain p(A=1|S=1)=371/436=0.85 , p(A=0|S=1)=65/436=0.15 , p(Y=1|A=1,S=1)=239/371=0.79 , p(Y=1|A=0,S=1)=53/65=0.82 , p(S=1|A=1)=371/473=0.78 , p(S=1|A=0)=65/103=0.63 , and ${\mathrm{RR}}_{S}=0.97$ .

**Figure 2.**
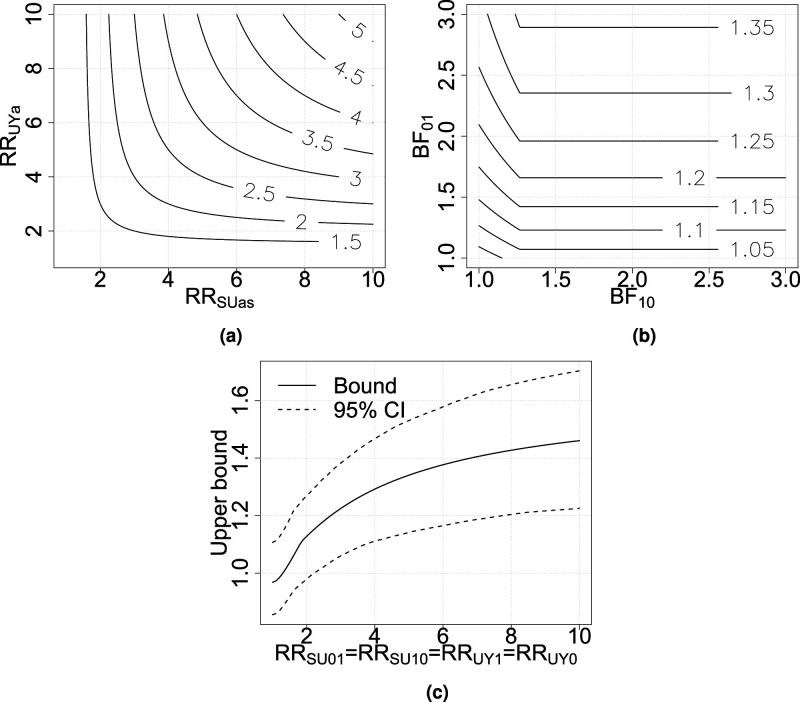
Contour plots for (a) ${\mathrm{BF}}_{as}$ , (b) the upper bound for ${\mathrm{CRR}}_{T}$ , and (c) 95% nonparametric confidence intervals for the sharp upper bound for ${\mathrm{CRR}}_{T}$ . BF: bias factor; CRR: causal risk ratio.

The contour plot in Figure 2(a) shows values of ${\mathrm{BF}}_{as}$ for different values of ${\mathrm{RR}}_{SU|as}$ and ${\mathrm{RR}}_{UY|a}$ , and the contour plot in Figure 2(b) shows values for the upper improved bound for ${\mathrm{CRR}}_{T}$ for different values on ${\mathrm{BF}}_{10}$ and ${\mathrm{BF}}_{01}$ . The plots can be used to determine an upper bound for ${\mathrm{CRR}}_{T}$ , the causal risk ratio of breakfast-eating on overweight for both married people/people living partners and single people/people not living with a partner, based on reasonable values for the sensitivity parameters. The lowest curve in Figure 2(b), visible in the bottom-left corner, is the bound for a null effect. The ‘‘crack” in the curves in Figure 2(b) indicates where ${\mathrm{BF}}_{10}=1/p(Y=1\;|\;A=1,S=1)$ .

Sampling variability should also be considered when calculating the bounds, since the probabilities are estimated. Here, we apply a nonparametric bootstrap resampling procedure. The probabilities used in the calculations of the bounds are sampled from binomial distributions where the parameters are taken from the data. A total of 1000 bootstrap samples are taken and the bounds are calculated. 95% confidence intervals for the upper bounds when the sensitivity parameters are assumed to be equal are calculated as the 0.025 and 0.975 quantiles for the bootstrap samples. The bounds and 95% point-wise confidence intervals are shown in Figure 2(c). The confidence intervals are fairly wide. The uncertainty is partly due to the relatively small sample size in the breakfast-skipping group. Furthermore, additional uncertainty that comes from generalizing the conclusions to the non-selected part of the population as well.

**Figure 3.**
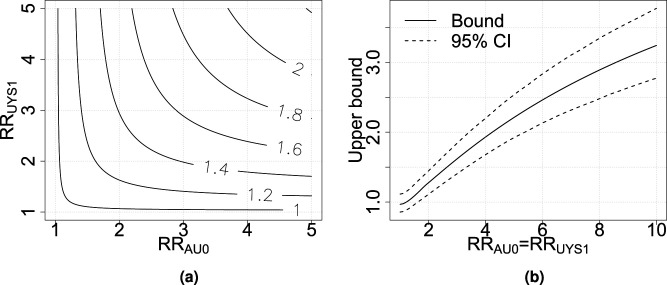
Contour plot for (a) the upper bound for ${\mathrm{CRR}}_{S}$ and (b) 95% nonparametric confidence intervals for the sharp upper bound for ${\mathrm{CRR}}_{S}$ . CRR: causal risk ratio.

Similarly, in Figure 3(a), the upper bound ${\mathrm{CRR}}_{S}$ is plotted for different values on the sensitivity parameters. Again, the plot can be used to determine an upper bound for ${\mathrm{CRR}}_{S}$ , the CRR of breakfast-eating on overweight for married people/people living partners, based on reasonable values for the sensitivity parameters. Sampling variability is considered using the same nonparametric bootstrap resampling procedure. The upper bounds and 95% point-wise confidence intervals are presented in Figure 3(b). The confidence intervals are not as wide as for the total population bounds. The sample size in the breakfast-skipping group is the same, but there is no additional uncertainty from generalizing the conclusions to the non-selected part of the population as it is only the selected part of the population that is of interest. Code for the calculations is found in Supplemental Appendix L.

## 6. Numerical example

**Figure 4.**
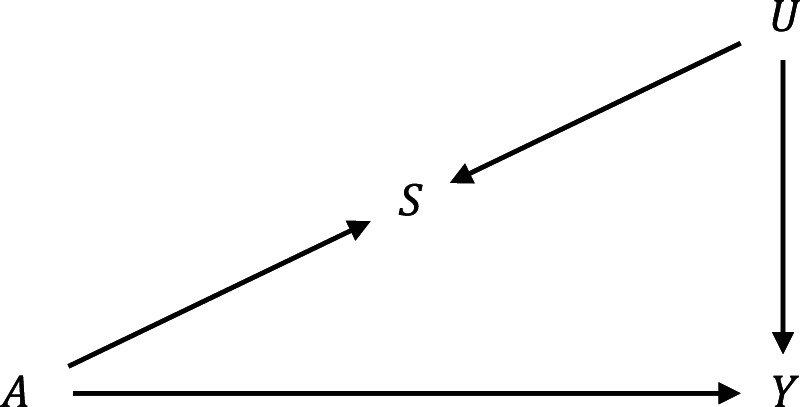
Structure of the data-generating process in the numerical example.

The performance of the SV and improved bounds for all four estimands are compared in a numerical example. The distributions are generated from the causal model in Figure 4 where Assumptions (2) to (4) hold. The model is parameterized as follows:

$$\begin{array}{rl} p(U=1) & =\mathrm{expit}(\theta_{1})\\ p(A=1) & =\mathrm{expit}(\theta_{2})\\ p(S=1\;|\;A,U) & =\mathrm{expit}(\alpha_{1}+\beta A+\gamma U+\delta AU)\\ p(Y=1\;|\;A,U) & =\mathrm{expit}(\alpha_{2}+\lambda A+\psi U+\zeta AU) \end{array}$$

where $\mathrm{expit}(x)=1/(1+e^{-x})$ is the inverse logit function. The coefficients β and γ for A , δ and λ for U and the interaction terms ψ and ζ are independently drawn from $N(0,\sigma^{2})$ , for σ=1 and σ=3 , respectively. The parameters $\theta_{1}$ , $\theta_{2}$ , $\alpha_{1}$ , and $\alpha_{2}$ are then set to obtain the two different marginal probabilities as follows:

$$\begin{array}{rl} p(U=1) & =0.20,0.50\\ p(E=1) & =0.05,0.20\\ p(S=1) & =0.50,0.80\\ p(Y=1) & =0.05,0.20 \end{array}$$

The reason for this setup is to compare the bounds for different distributions and different causal effects, while keeping the marginal probabilities to reasonable values. The different values of the standard deviations determines how likely large causal effects and strong selection dependencies are.

The setup results in 32 combinations of probabilities and standard deviations. For each combination, 1000 distributions are generated, and for each distribution, the causal estimand, the observed estimand, and the SV and improved lower and upper bounds are calculated using the true probabilities. For the total population estimands, the alternative bounds which sets p(S=1|A=a)=0 , are also calculated. The bounds are evaluated using two measures. First, the proportion, p , when SV’s bounds are equal to the sharp bound. This measure is not of interest for the comparisons between the sharp and SV bounds for the total population estimands and the risk difference in the subpopulation as the SV bounds for these estimands are not sharp since the probabilities are not equal to 0. Second, the absolute mean distance between the causal estimand and the bounds, Δ . The bounds are compared when the true sensitivity parameters are used. The results for σ=3 are presented in Supplemental Appendix K.

In Table 1, the results for the sharp bounds for the risk ratio in the total population are presented. Here, Δ is approximately double for the SV bound compared to the sharp bound. However, the sharp bounds use the selection probabilities p(S=1|A=a) which the SV bounds do not. When a comparison is made between the SV bounds and the alternative bounds with p(S=1|A=a)=0 , Table 2, the results instead are very similar for the two bounds, which is not surprising as the alternative bound is equal to the SV bound in specific regions. However, all bounds are quite conservative as the distance between the bounds and the causal estimand is multiple times larger than the size of the causal estimand.

**Table 1.** Results for ${\mathrm{CRR}}_{T}$ with σ=1 . $p_{L}$ and $p_{U}$ are the proportions that SV’s lower and upper bounds are equal to the sharp lower and upper bounds. $\Delta_{L}^{\text{sharp}}$ , $\Delta_{U}^{\text{sharp}}$ , $\Delta_{L}^{\text{SV}}$ , and $\Delta_{U}^{\text{SV}}$ are the mean distance between $\log\;{\mathrm{CRR}}_{T}$ and the logarithm of bounds. ${\mathrm{CRR}}_{T}$ is the logarithm of the causal estimand.

p(U=1)	p(A=1)	p(Y=1)	p(S=1)	$p_{L}$	$p_{U}$	$\Delta_{L}^{\text{sharp}}$	$\Delta_{U}^{\text{sharp}}$	$\Delta_{L}^{\text{SV}}$	$\Delta_{U}^{\text{SV}}$	${\mathrm{CRR}}_{T}$
0.20	0.05	0.05	0.50	0	0	0.18	0.20	0.41	0.40	0.06
0.20	0.05	0.05	0.80	0	0	0.08	0.10	0.41	0.39	0.04
0.20	0.05	0.20	0.50	0	0	0.17	0.18	0.37	0.35	−0.02
0.20	0.05	0.20	0.80	0	0	0.07	0.07	0.37	0.34	−0.06
0.20	0.20	0.05	0.50	0	0	0.19	0.20	0.43	0.40	0.03
0.20	0.20	0.05	0.80	0	0	0.07	0.09	0.42	0.39	0.06
0.20	0.20	0.20	0.50	0	0	0.16	0.18	0.35	0.36	−0.07
0.20	0.20	0.20	0.80	0	0	0.08	0.08	0.38	0.35	−0.04
0.50	0.05	0.05	0.50	0	0	0.21	0.21	0.46	0.43	0.03
0.50	0.05	0.05	0.80	0	0	0.09	0.08	0.44	0.39	0.10
0.50	0.05	0.20	0.50	0	0	0.16	0.18	0.37	0.37	−0.05
0.50	0.05	0.20	0.80	0	0	0.08	0.08	0.37	0.34	−0.01
0.50	0.20	0.05	0.50	0	0	0.20	0.20	0.46	0.43	0.08
0.50	0.20	0.05	0.80	0	0	0.09	0.09	0.45	0.40	0.08
0.50	0.20	0.20	0.50	0	0	0.17	0.16	0.38	0.35	−0.01
0.50	0.20	0.20	0.80	0	0	0.07	0.08	0.39	0.36	−0.01

CRR: causal risk ratio; SV: Smith and VanderWeele.

**Table 2.** Results for ${\mathrm{CRR}}_{T}$ with σ=1 . $p_{L}$ and $p_{U}$ are the proportions that SV’s lower and upper bounds are equal to the alternative lower and upper bounds. $\Delta_{L}^{\text{alt}}$ , $\Delta_{U}^{\text{alt}}$ , $\Delta_{L}^{\text{SV}}$ , and $\Delta_{U}^{\text{SV}}$ are the mean distance between $\log\;{\mathrm{CRR}}_{T}$ and the logarithm of bounds. ${\mathrm{CRR}}_{T}$ is the logarithm of the causal estimand.

p(U=1)	p(A=1)	p(Y=1)	p(S=1)	$p_{L}$	$p_{U}$	$\Delta_{L}^{\text{alt}}$	$\Delta_{U}^{\text{alt}}$	$\Delta_{L}^{\text{SV}}$	$\Delta_{U}^{\text{SV}}$	${\mathrm{CRR}}_{T}$
0.20	0.05	0.05	0.50	1	1	0.41	0.40	0.41	0.40	0.06
0.20	0.05	0.05	0.80	1	1	0.41	0.39	0.41	0.39	0.04
0.20	0.05	0.20	0.50	1	0.99	0.37	0.35	0.37	0.35	−0.02
0.20	0.05	0.20	0.80	1	0.99	0.37	0.34	0.37	0.34	−0.06
0.20	0.20	0.05	0.50	1	1	0.43	0.40	0.43	0.40	0.03
0.20	0.20	0.05	0.80	1	1	0.42	0.39	0.42	0.39	0.06
0.20	0.20	0.20	0.50	1	0.99	0.35	0.36	0.35	0.36	−0.07
0.20	0.20	0.20	0.80	1	0.99	0.38	0.35	0.38	0.35	−0.04
0.50	0.05	0.05	0.50	1	1	0.46	0.43	0.46	0.43	0.03
0.50	0.05	0.05	0.80	1	1	0.44	0.39	0.44	0.39	0.10
0.50	0.05	0.20	0.50	1	0.99	0.37	0.37	0.37	0.37	−0.05
0.50	0.05	0.20	0.80	1	1	0.37	0.34	0.37	0.34	−0.01
0.50	0.20	0.05	0.50	1	1	0.46	0.42	0.46	0.43	0.08
0.50	0.20	0.05	0.80	1	1	0.45	0.40	0.45	0.40	0.08
0.50	0.20	0.20	0.50	1	1	0.38	0.35	0.38	0.35	−0.01
0.50	0.20	0.20	0.80	1	1	0.39	0.36	0.39	0.36	−0.01

CRR: causal risk ratio; SV: Smith and VanderWeele.

The results for the sharp bounds for the risk difference in the total population are similar to the risk ratio, see Table 3. The SV bounds for the risk difference in the total population are rather conservative, and these results are in line with previous results.^18^ Here, Δ is much smaller for the sharp bound than for the SV bound. Furthermore, Δ is about the same size as the causal estimand. The results for the alternative bounds with p(S=1|A=a)=0 are similar to those for the sharp bounds, Table 4.

**Table 3.** Results for ${\mathrm{CRD}}_{T}$ with σ=1 . $p_{L}$ and $p_{U}$ are the proportions that SV’s lower and upper bounds are equal to the sharp lower and upper bounds. $\Delta_{L}^{\text{sharp}}$ , $\Delta_{U}^{\text{sharp}}$ , $\Delta_{L}^{\text{SV}}$ , and $\Delta_{U}^{\text{SV}}$ are the mean distance between ${\mathrm{CRD}}_{T}$ and the bounds. ${\mathrm{CRD}}_{T}$ is the causal estimand.

p(U=1)	p(A=1)	p(Y=1)	p(S=1)	$p_{L}$	$p_{U}$	$\Delta_{L}^{\text{sharp}}$	$\Delta_{U}^{\text{sharp}}$	$\Delta_{L}^{\text{SV}}$	$\Delta_{U}^{\text{SV}}$	${\mathrm{CRD}}_{T}$
0.20	0.05	0.05	0.50	0	0	0.01	0.02	1.37	1.24	0.02
0.20	0.05	0.05	0.80	0	0	0.00	0.02	1.38	1.25	0.02
0.20	0.05	0.20	0.50	0	0	0.03	0.04	1.36	1.29	0.04
0.20	0.05	0.20	0.80	0	0	0.01	0.02	1.40	1.30	0.04
0.20	0.20	0.05	0.50	0	0	0.01	0.01	1.40	1.34	0.01
0.20	0.20	0.05	0.80	0	0	0.00	0.01	1.44	1.25	0.01
0.20	0.20	0.20	0.50	0	0	0.03	0.04	1.35	1.27	0.02
0.20	0.20	0.20	0.80	0	0	0.01	0.02	1.39	1.31	0.02
0.50	0.05	0.05	0.50	0	0	0.01	0.02	1.37	1.26	0.03
0.50	0.05	0.05	0.80	0	0	0.01	0.01	1.41	1.28	0.03
0.50	0.05	0.20	0.50	0	0	0.03	0.04	1.32	1.31	0.05
0.50	0.05	0.20	0.80	0	0	0.02	0.02	1.36	1.32	0.05
0.50	0.20	0.05	0.50	0	0	0.01	0.01	1.40	1.24	0.02
0.50	0.20	0.05	0.80	0	0	0.00	0.01	1.46	1.28	0.02
0.50	0.20	0.20	0.50	0	0	0.03	0.04	1.34	1.28	0.04
0.50	0.20	0.20	0.80	0	0	0.01	0.02	1.39	1.32	0.04

CRD: causal risk difference; SV: Smith and VanderWeele.

**Table 4.** Results for ${\mathrm{CRD}}_{T}$ with σ=1 . $p_{L}$ and $p_{U}$ are the proportions that SV’s lower and upper bounds are equal to the alternative lower and upper bounds. $\Delta_{L}^{\text{alt}}$ , $\Delta_{U}^{\text{alt}}$ , $\Delta_{L}^{\text{SV}}$ , and $\Delta_{U}^{\text{SV}}$ are the mean distance between ${\mathrm{CRD}}_{T}$ and the bounds. ${\mathrm{CRD}}_{T}$ is the causal estimand.

p(U=1)	p(A=1)	p(Y=1)	p(S=1)	$p_{L}$	$p_{U}$	$\Delta_{L}^{\text{alt}}$	$\Delta_{U}^{\text{alt}}$	$\Delta_{L}^{\text{SV}}$	$\Delta_{U}^{\text{SV}}$	${\mathrm{CRD}}_{T}$
0.20	0.05	0.05	0.50	0	0	0.02	0.03	1.37	1.24	0.02
0.20	0.05	0.05	0.80	0	0	0.02	0.03	1.38	1.25	0.02
0.20	0.05	0.20	0.50	0	0	0.07	0.08	1.36	1.29	0.04
0.20	0.05	0.20	0.80	0	0	0.07	0.07	1.40	1.30	0.04
0.20	0.20	0.05	0.50	0	0	0.02	0.07	1.40	1.34	0.01
0.20	0.20	0.05	0.80	0	0	0.02	0.02	1.44	1.25	0.01
0.20	0.20	0.20	0.50	0	0	0.07	0.08	1.35	1.27	0.02
0.20	0.20	0.20	0.80	0	0	0.07	0.08	1.39	1.31	0.02
0.50	0.05	0.05	0.50	0	0	0.03	0.03	1.37	1.26	0.03
0.50	0.05	0.05	0.80	0	0	0.03	0.03	1.41	1.28	0.03
0.50	0.05	0.20	0.50	0	0	0.08	0.09	1.32	1.31	0.05
0.50	0.05	0.20	0.80	0	0	0.07	0.08	1.36	1.32	0.05
0.50	0.20	0.05	0.50	0	0	0.02	0.03	1.40	1.24	0.02
0.50	0.20	0.05	0.80	0	0	0.02	0.02	1.46	1.28	0.02
0.50	0.20	0.20	0.50	0	0	0.07	0.08	1.34	1.28	0.04
0.50	0.20	0.20	0.80	0	0	0.07	0.08	1.39	1.32	0.04

CRD: causal risk difference; SV: Smith and VanderWeele.

For the risk ratio in the subpopulation, Table 5, the results are very different. Here, the SV bounds are always equal to the sharp bounds and Δ are thus the same for the two bounds. Furthermore, Δ is about the same size as the causal estimand. The results for the risk difference in the subpopulation, see Table 6, are similar to the results for the risk ratio in the subpopulation; Δ is often the same for the two bounds, when rounded to two decimals, and Δ is about the same size as the estimand, which again indicates that the bounds are not too conservative.

**Table 5.** Results for ${\mathrm{CRR}}_{S}$ with σ=1 . $p_{L}$ and $p_{U}$ are the proportions that SV’s lower and upper bounds are equal to the sharp lower and upper bounds. $\Delta_{L}^{\text{sharp}}$ , $\Delta_{U}^{\text{sharp}}$ , $\Delta_{L}^{\text{SV}}$ , and $\Delta_{U}^{\text{SV}}$ are the mean distance between $\log\;{\mathrm{CRR}}_{S}$ and the logarithm of bounds. ${\mathrm{CRR}}_{S}$ is the logarithm of the causal estimand.

p(U=1)	p(A=1)	p(Y=1)	p(S=1)	$p_{L}$	$p_{U}$	$\Delta_{L}^{\text{sharp}}$	$\Delta_{U}^{\text{sharp}}$	$\Delta_{L}^{\text{SV}}$	$\Delta_{U}^{\text{SV}}$	${\mathrm{CRR}}_{S}$
0.20	0.05	0.05	0.50	1	1	0.11	0.15	0.11	0.15	0.06
0.20	0.05	0.05	0.80	1	1	0.06	0.10	0.06	0.10	0.04
0.20	0.05	0.20	0.50	1	1	0.10	0.13	0.10	0.13	−0.03
0.20	0.05	0.20	0.80	1	1	0.05	0.06	0.05	0.06	−0.06
0.20	0.20	0.05	0.50	1	1	0.11	0.14	0.11	0.14	0.03
0.20	0.20	0.05	0.80	1	1	0.05	0.07	0.05	0.07	0.06
0.20	0.20	0.20	0.50	1	1	0.09	0.13	0.09	0.13	−0.07
0.20	0.20	0.20	0.80	1	1	0.06	0.07	0.06	0.07	−0.04
0.50	0.05	0.05	0.50	1	1	0.13	0.14	0.13	0.14	0.03
0.50	0.05	0.05	0.80	1	1	0.06	0.07	0.06	0.07	0.10
0.50	0.05	0.20	0.50	1	1	0.10	0.13	0.10	0.13	−0.04
0.50	0.05	0.20	0.80	1	1	0.06	0.07	0.06	0.07	−0.01
0.50	0.20	0.05	0.50	1	1	0.13	0.14	0.13	0.14	0.08
0.50	0.20	0.05	0.80	1	1	0.06	0.07	0.06	0.07	0.08
0.50	0.20	0.20	0.50	1	1	0.10	0.12	0.10	0.12	−0.02
0.50	0.20	0.20	0.80	1	1	0.05	0.07	0.05	0.07	−0.01

CRR: causal risk ratio; SV: Smith and VanderWeele.

**Table 6.** Results for ${\mathrm{CRD}}_{S}$ with σ=1 . $p_{L}$ and $p_{U}$ are the proportions that SV’s lower and upper bounds are equal to the sharp lower and upper bounds. $\Delta_{L}^{\text{sharp}}$ , $\Delta_{U}^{\text{sharp}}$ , $\Delta_{L}^{\text{SV}}$ , and $\Delta_{U}^{\text{SV}}$ are the mean distance between ${\mathrm{CRD}}_{S}$ and the bounds. ${\mathrm{CRD}}_{S}$ is the causal estimand.

p(U=1)	p(A=1)	p(Y=1)	p(S=1)	$p_{L}$	$p_{U}$	$\Delta_{L}^{\text{sharp}}$	$\Delta_{U}^{\text{sharp}}$	$\Delta_{L}^{\text{SV}}$	$\Delta_{U}^{\text{SV}}$	${\mathrm{CRD}}_{S}$
0.20	0.05	0.05	0.50	0	0	0.01	0.01	0.01	0.01	0.02
0.20	0.05	0.05	0.80	0	0	0.00	0.01	0.00	0.01	0.02
0.20	0.05	0.20	0.50	0	0	0.02	0.04	0.03	0.04	0.04
0.20	0.05	0.20	0.80	0	0	0.01	0.02	0.01	0.02	0.04
0.20	0.20	0.05	0.50	0	0	0.01	0.01	0.01	0.01	0.01
0.20	0.20	0.05	0.80	0	0	0.00	0.00	0.00	0.01	0.02
0.20	0.20	0.20	0.50	0	0	0.02	0.03	0.02	0.04	0.02
0.20	0.20	0.20	0.80	0	0	0.01	0.02	0.01	0.02	0.02
0.50	0.05	0.05	0.50	0	0	0.01	0.01	0.01	0.01	0.03
0.50	0.05	0.05	0.80	0	0	0.00	0.01	0.01	0.01	0.03
0.50	0.05	0.20	0.50	0	0	0.02	0.03	0.03	0.04	0.05
0.50	0.05	0.20	0.80	0	0	0.02	0.02	0.02	0.02	0.05
0.50	0.20	0.05	0.50	0	0	0.01	0.01	0.01	0.01	0.02
0.50	0.20	0.05	0.80	0	0	0.00	0.00	0.00	0.01	0.02
0.50	0.20	0.20	0.50	0	0	0.02	0.03	0.03	0.04	0.03
0.50	0.20	0.20	0.80	0	0	0.01	0.02	0.01	0.02	0.04

CRD: causal risk difference; SV: Smith and VanderWeele.

The results for σ=3 , seen in Tables 8 to 13 in Supplemental Appendix K, are similar to σ=1 but with more variation, especially for the upper bound for ${\mathrm{CRR}}_{S}$ , where the sharp bounds in some instances are tighter than the SV bound (Table 12).

## 7. Discussion

Bounds for bias are one type of sensitivity analysis. Here, we add to the literature on bounds for causal estimands under selection bias in similar ways as Sjölander^8^ did for bounds for causal estimands under confounding. First, we have derived new properties of the previously proposed SV bounds. We have shown that the sensitivity parameters are variation independent, which is important when considering which values to set the sensitivity parameters to. Furthermore, we have also investigated the sharpness of the SV bounds. The SV bounds are sharp under certain conditions for the data distribution and sensitivity parameters, some of which are more likely to be fulfilled than others.

Since the SV bounds are only sharp under certain conditions, improved bounds can be derived. Using the same sensitivity parameters, we have derived improved bounds which are sharp. The bounds for the causal estimands in the total population require additional information on the selection probability compared to the SV bounds. In some studies, for example, register-based studies where data is available on all subjects, including the non-selected ones, these probabilities are available. In other studies, they are unknown. If this knowledge is not available, alternative bounds can be calculated by setting these probabilities to zero. The alternative bounds are equal to the SV bounds in some regions and tighter in others. The improved bounds for the causal estimands in the subpopulation is simply the minimum value of the SV bound and the sharp limit. Thus, the improved bounds are equal to the SV bounds when the latter are in the sharp region and tighter when they are not.

There are some limitations to the bounds presented here. The sensitivity parameters of the bounds are defined as ratios between the maximum and minimum of conditional probabilities. In the case of many selection variables, as is not uncommon in practical studies, the sensitivity parameters can get very large, which results in bounds that too conservative to give any information about the size of the bias. Furthermore, the results in this article are derived given fixed values of the sensitivity parameters. Determining a reasonable range of the sensitivity parameters by calibrating them against observed quantities is an important but technically difficult topic for future research. Lastly, the bounds are derived under the assumption of no unmeasured confounding. However, it is possible that a study suffers from several types of biases. An important contribution would therefore be a sensitivity analysis that takes multiple types of biases into account.

## Supplemental Material

sj-pdf-1-smm-10.1177_09622802251374168 - Supplemental material for Investigations of sharp bounds for causal effects under selection bias Supplemental material, sj-pdf-1-smm-10.1177_09622802251374168 for Investigations of sharp bounds for causal effects under selection bias by Stina Zetterstrom, Arvid Sjölander and Ingeborg Waernbaum in Statistical Methods in Medical Research

## References

[1] Flanders WD Ye D . Limits for the magnitude of M-bias and certain other types of structural selection bias. Epidemiology 2019; 30: 501–508. 31033689 10.1097/EDE.0000000000001031

[2] Greenland S . Quantifying biases in causal models: classical confounding vs collider-stratification bias. Epidemiology 2003; 14: 300–306. 12859030

[3] Huang TH Lee WC . Bounding formulas for selection bias. Am J Epidemiol 2015; 182: 868–872. 26519426 10.1093/aje/kwv130

[4] Sjölander A . Selection bias with outcome-dependent sampling. Epidemiology 2023; 34: 186–191. 36722800 10.1097/EDE.0000000000001567

[5] Zetterstrom S Waernbaum I . Selection bias and multiple inclusion criteria in observational studies. Epidemiol Method 2022; 11: 20220108.

[6] Duarte G Finkelstein N Knox D , et al. An automated approach to causal inference in discrete settings. J Am Stat Assoc 2024; 119: 1778–1793. 39553407 10.1080/01621459.2023.2216909 PMC11566246

[7] Smith L VanderWeele T . Bounding bias due to selection. Epidemiology 2019; 30: 509–516. 31033690 10.1097/EDE.0000000000001032 PMC6553568

[8] Sjölander A . A note on a sensitivity analysis for unmeasured confounding, and the related e-value. J Causal Inference 2020; 8: 229–248.

[9] Neyman J . On the application of probability theory to agricultural experiments, essay on principles. Roczniki nauk rolczych X, 1-51. In Polish English translation by D.M. Dabrowska and T.P. Speed. Stat Sci 1923; 5: 465–472.

[10] Rubin DB . Estimating causal effects of treatments in randomized and nonrandomized studies. J Educ Psychol 1974; 66: 688–701.

[11] Song WO Chun OK Obayashi S , et al. Is consumption of breakfast associated with body mass index in US adults? J Am Diet Assoc 2005; 105: 1373–1382. 16129078 10.1016/j.jada.2005.06.002

[12] Centers for Disease Control and Prevention (CDC) National Center for Health Statistics (NCHS) (1999–2018) National health and nutrition examination survey data. https://wwwn.cdc.gov/nchs/nhanes/default.aspx.

[13] Chen HY . A semiparametric odds ratio model for measuring association. Biometrics 2007; 63: 413–421. 17688494 10.1111/j.1541-0420.2006.00701.x

[14] Nabi R Bhattacharya R Shpitser I . Full law identification in graphical models of missing data: completeness results. In: *International conference on machine learning*, 2020, pp.7153–7163. PMLR. PMC7716645 33283197

[15] Malinsky D Shpitser I Tchetgen EJT . Semiparametric inference for nonmonotone missing-not-at-random data: the no self-censoring model. J Am Stat Assoc 2022; 117: 1415–1423. 36246417 10.1080/01621459.2020.1862669 PMC9562456

[16] Shpitser I . The Lauritzen-Chen likelihood for graphical models. In: *International conference on artificial intelligence and statistics*, 2023, pp.4181–4195. PMLR.

[17] Ding P VanderWeele TJ . Sensitivity analysis without assumptions. Epidemiology 2016; 27: 368–377. 26841057 10.1097/EDE.0000000000000457 PMC4820664

[18] Zetterstrom S . Bounds for selection bias using outcome probabilities. Epidemiol Method 2024; 13: 20230033.

