# Supplementary material for: Investigations of sharp bounds for causal effects under selection bias
Source: Stat Methods Med Res. 2025 Sep 30;34(12):2270–83. doi: 10.1177/09622802251374168 (PMC12669388; doi:10.1177/09622802251374168)
Supplement: sj-pdf-1-smm-10.1177_09622802251374168 - Supplemental material for Investigations of sharp bounds for causal effects under selection bias [file sj-pdf-1-smm-10.1177_09622802251374168.pdf]

Journal Title
XX(X):1–33
©The Author(s) 2025
Reprints and permission:
sagepub.co.uk/journalsPermissions.nav
DOI: 10.1177/ToBeAssigned
www.sagepub.com/
SAGE

Supplemental Appendix

Stina Zetterstrom[1], Arvid Sjölander[2] and Ingeborg Waernbaum[1]

A Summary of notation

The notation is summarized in Table 1.

B Causal estimands

Under (1), (2) and (3), we can rewrite $p(Y^a = y)$ as

$$\begin{aligned} p(Y^a = y) &= \mathrm{E}_U(p(Y^a = y \mid U)) \\ &= \mathrm{E}_U(p(Y^a = y \mid A = a, U)) \\ &= \mathrm{E}_U(p(Y = y \mid A = a, U)) \\ &= \mathrm{E}_U(p(Y = y \mid S = 1, A = a, U)), \end{aligned}$$

where the first equality follows from the law of total probability, the second from (2), the third from (1), and the fourth from (3).

Under (1) and (4), we can rewrite $p(Y^a = y \mid S = 1)$ as

$$\begin{aligned} p(Y^a = y \mid S = 1) &= \mathrm{E}_U(p(Y^a = y \mid S = 1, U) \mid S = 1) \\ &= \mathrm{E}_U(p(Y^a = y \mid S = 1, A = a, U) \mid S = 1) \\ &= \mathrm{E}_U(p(Y = y \mid S = 1, A = a, U) \mid S = 1), \end{aligned}$$

where the first equality follows from the law of total probability, the second from (4), and the third from (1).

[1]Uppsala University, Sweden
[2]Karolinska Institute, Sweden

Corresponding author:
Stina Zetterstrom, Department of Statistics, Uppsala University Box 513, 75120, Uppsala, Sweden, +46706069133
Email: stina.zetterstrom@gmail.com

Table 1. Summary of the notation.

Symbol	Explanation
Y^a	Potential outcome
A	Exposure variable
Y	Outcome variable
S	Selection indicator variable
X	Pre-exposure covariate
U	Unmeasured (set of) variable
X	Pre-exposure covariate
$p(\cdot)$	Probability/distribution
RD_S	Risk difference
RR_S	Risk ratio
CRD_T	Causal risk difference in the total population
CRR_T	Causal risk ratio in the total population
CRD_S	Causal risk difference in the subpopulation
CRR_S	Causal risk ratio in the subpopulation
$\mathcal{B}_{\text{RR}_T}$	Selection bias for the risk ratio in the total subpopulation
$\mathcal{B}_{\text{RD}_T}$	Selection bias for the risk difference in the total subpopulation
$\mathcal{B}_{\text{RR}_T}$	Selection bias for the risk ratio in the total subpopulation
LB_{CE}	Lower bound for the causal estimand
UB_{CE}	Upper bound for the causal estimand
LB_{B}	Lower bound for the selection bias
UB_{B}	Upper bound for the selection bias
$\text{RR}_{SU\|as}$	Sensitivity parameter for the total population bounds
$\text{RR}_{UY\|a}$	Sensitivity parameter for the total population bounds
$\text{RR}_{AU\|a}$	Sensitivity parameter for the subpopulation bounds
$\text{RR}_{UY\|S=1}$	Sensitivity parameter for the subpopulation bounds
BF_{as}	Bias factor for the total population bounds
BF_a	Bias factor for the subpopulation bounds
l_a	Lower bounds for $p(Y^a = 1)$
u_a	Upper bounds for $p(Y^a = 1)$
l'_a	Lower bounds for $p(Y^a = 1\|S = 1)$
u'_a	Upper bounds for $p(Y^a = 1\|S = 1)$

C An example where the independence in (2) holds but the independence in (4) does not

SV used the causal diagram in Figure 1 (their Figure C) to illustrate the obesity paradox. Here, A is obesity, S is coronary artery disease (CAD), Y is 1-year risk of death, and U is the set of all (measured and unmeasured) factors influencing CAD and mortality. They used their results to make inference on the causal risk ratio in the selected subpopulation, thus effectively assuming that the independence in (4) holds. However, it can be shown that, although the independence in (2) holds under the causal diagram in Figure 1, the independence in (4) does not.

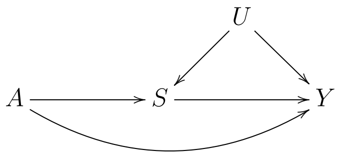

Figure 1. Causal diagram borrowed from SV.

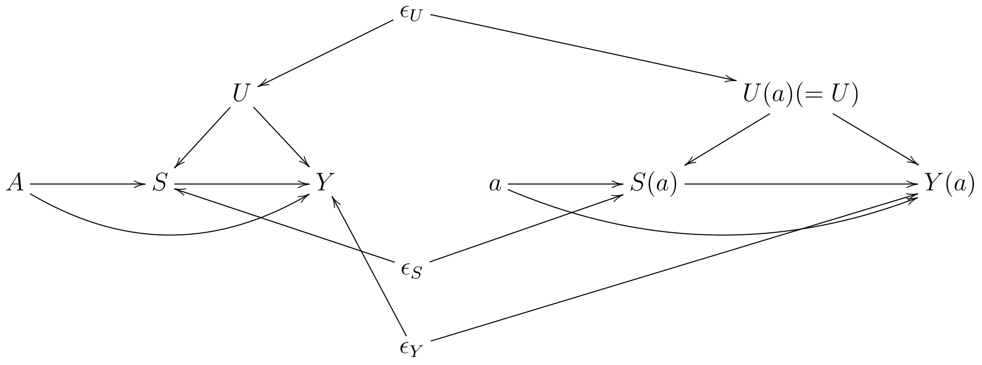

Figure 2. Twin network (Balke and Pearl 1994) connecting the factual world where A varies randomly (left panel) to the counterfactual world where A is set to a (right panel).

To see this, consider the causal diagram in Figure 2. In this figure, the left part corresponds to the factual world where A varies randomly, and the right part corresponds to the counterfactual world where A is set to the fixed value a. The two worlds are connected via the exogenous 'error terms' ϵ_U, ϵ_S and ϵ_Y, which have the same values in both worlds. From Figure 2 we see that the independence in (2) holds since A and Y^a are d-separated by U. However, (4) does not hold since A and Y^a are not d-separated by (S, U). This is because conditioning on $S = 1$ opens the path $A \rightarrow S \leftarrow \epsilon_S \rightarrow S(a) \rightarrow Y^a$ on which S is a collider.

D Proof of Theorem 1

In this theorem we show that the sensitivity parameters are restricted by 1 from their definition and that they are variationally independent. The latter is shown by fixing the parameters and then show that there always exists a joint distribution that marginalizes to these parameters, or in other words, that there exists a well-defined joint distribution that gives these sensitivity parameters.

Since $\mathrm{RR}_{UY|a} = \max_u p(Y = 1 \mid A = a, U = u)/\min_u p(Y = 1 \mid A = a, U = u)$, $a \in \{0, 1\}$, $\mathrm{RR}_{UY|a} \geq 1$, $a\{0, 1\}$ by definition. Define $g(u) = p(U = u \mid A = 1, S = 1)/p(U = u \mid A = 1, S = 0)$. Note that $\mathrm{E}[g(U) \mid A = 1, S = 0] = \sum_u g(u)p(U = u \mid A = 1, S = 0) = \sum_u p(U = u \mid A = 1, S =$

$1) = 1$, which gives that $\mathrm{RR}_{SU|11} = \max_u g(u) \geq 1$. Similar arguments gives $\mathrm{RR}_{SU|10} \geq 1$, $\mathrm{RR}_{SU|01} \geq 1$, and $\mathrm{RR}_{SU|00} \geq 1$.

To show that $\{p(Y, A \mid S = 1),\ p(S = 1 \mid A = 1),\ p(S = 1 \mid A = 0),\ \mathrm{RR}_{SU|00},\ \mathrm{RR}_{SU|01},\ \mathrm{RR}_{SU|10},\ \mathrm{RR}_{SU|11},\ \mathrm{RR}_{UY|0},\ \mathrm{RR}_{UY|1}\}$ are variation independent, we show that it is possible to construct a distribution $p(Y, A, S, U)$ for which Assumption (3) holds, and which marginalizes to any given set $\{p^*(Y, A \mid S = 1),\ p^*(S = 1 \mid A = 1),\ p^*(S = 1 \mid A = 0),\ \mathrm{RR}^*_{SU|00},\ \mathrm{RR}^*_{SU|01},\ \mathrm{RR}^*_{SU|10},\ \mathrm{RR}^*_{SU|11},\ \mathrm{RR}^*_{UY|0},\ \mathrm{RR}^*_{UY|1}\}$. We construct the distribution $p(Y, A, S, U)$ in the following steps.

1. Define $p^*_a = p^*(S = 1 \mid A = a)$, $q^*_1 = p^*(A = 1 \mid S = 1)$. Set

$$p(S = 1) = \frac{p^*_0 p^*_1}{p^*_0 p^*_1 + p^*_0(1 - p^*_1)q^*_1 + p^*_1(1 - p^*_0)(1 - q^*_1)},$$

$$p(A = 1 \mid S = 0) = \frac{p^*_0(1 - p^*_1)q^*_1}{p^*_0(1 - p^*_1)q^*_1 + p^*_1(1 - p^*_0)(1 - q^*_1)}$$

and

$$p(A = 1 \mid S = 1) = q^*_1.$$

We have that $0 < \{p(S = 1), p(A = 1 \mid S = 0), p(A = 1 \mid S = 1)\} < 1$. From Bayes' rule we also have that $p(S = 1 \mid A = a) = p^*_a$.

2. Let U be ternary, with

$$\begin{aligned}
p(U = 0 \mid A = a, S = 0) &= \frac{(1 - \epsilon_a)\mathrm{RR}^*_{SU|a0}(\mathrm{RR}^*_{SU|a1} - 1)}{\mathrm{RR}^*_{SU|a0}\mathrm{RR}^*_{SU|a1} - 1} \\
p(U = 1 \mid A = a, S = 0) &= \frac{(1 - \epsilon_a)(\mathrm{RR}^*_{SU|a0} - 1)}{\mathrm{RR}^*_{SU|a0}\mathrm{RR}^*_{SU|a1} - 1} \\
p(U = 2 \mid A = a, S = 0) &= \epsilon_a \\
p(U = 0 \mid A = a, S = 1) &= \frac{(1 - \epsilon_a)(\mathrm{RR}^*_{SU|a1} - 1)}{\mathrm{RR}^*_{SU|a0}\mathrm{RR}^*_{SU|a1} - 1} \\
p(U = 1 \mid A = a, S = 1) &= \frac{(1 - \epsilon_a)\mathrm{RR}^*_{SU|a1}(\mathrm{RR}^*_{SU|a0} - 1)}{\mathrm{RR}^*_{SU|a0}\mathrm{RR}^*_{SU|a1} - 1} \\
p(U = 2 \mid A = a, S = 1) &= \epsilon_a,
\end{aligned}$$

where $0 < \epsilon_a < 1$ is defined in the next step. We have that $\sum_k p(U = k \mid A = a, S = s) = 1$ for $(a, s) \in \{0, 1\}$ and $0 < p(U = k \mid A = a, S = s) < 1$ for $k \in \{0, 1, 2\}$ and $(a, s) \in \{0, 1\}$. We further have that $p(U = 0 \mid A = a, S = 1)/p(U = 0 \mid A = a, S = 0) = 1/\mathrm{RR}^*_{SU|a0}$, $p(U = 1 \mid A = a, S = 1)/p(U = 1 \mid A = a, S = 0) = \mathrm{RR}^*_{SU|a1}$ and $p(U = 2 \mid A = a, S = 1)/p(U = 2 \mid A = a, S = 0) = 1$, so that $\mathrm{RR}_{SU|a0} = p(U = 0 \mid A = a, S = 0)/p(U = 0 \mid A = a, S = 1) = \mathrm{RR}^*_{SU|a0}$ and $\mathrm{RR}_{SU|a1} = p(U = 1 \mid A = a, S = 1)/p(U = 1 \mid A = a, S = 0) = \mathrm{RR}^*_{SU|a1}$.

3. Set

$$
\begin{aligned}
p(Y=1\,|\,A=a,U=0,S=s) &= p^*(Y=1\,|\,A=a,S=1)\\
p(Y=1\,|\,A=a,U=1,S=s) &= p^*(Y=1\,|\,A=a,S=1)\frac{\mathrm{RR}^*_{UY|a}\{p(U=1\,|\,A=a,S=1)+\epsilon_a\}}{\mathrm{RR}^*_{UY|a}p(U=1\,|\,A=a,S=1)+\epsilon_a}\\
p(Y=1\,|\,A=a,U=2,S=s) &= p^*(Y=1\,|\,A=a,S=1)\frac{p(U=1\,|\,A=a,S=1)+\epsilon_a}{\mathrm{RR}^*_{UY|a}p(U=1\,|\,A=a,S=1)+\epsilon_a}
\end{aligned}
$$

We have that

$$
\begin{aligned}
&\frac{\partial p(Y=1\,|\,A=a,U=1,S=s)}{\partial \epsilon_a}\\
&\quad = p^*(Y=1\,|\,A=a,S=1)\mathrm{RR}^*_{UY|a}\frac{p(U=1\,|\,A=a,S=1)(\mathrm{RR}^*_{UY|a}-1)}{\{\mathrm{RR}^*_{UY|a}p(U=1\,|\,A=a,S=1)+\epsilon_a\}^2} > 0,
\end{aligned}
$$

which implies that $p(Y=1\,|\,A=a,U=1,S=1)$ is increasing in ϵ_a, with $\lim_{\epsilon_a\to 0} p(Y=1\,|\,A=a,U=1,S=1) = p^*(Y=1\,|\,A=a,S=1)$. Hence, we let ϵ_a be an arbitrary number between 0 and 1, sufficiently close to 0 to ensure that $p(Y=1\,|\,A=a,U=1,S=1)<1$. We thus have that $0<p(Y=1\,|\,A=a,U=k,S=s)<1$ for $(a,s)\in\{0,1\}$ and $k\in\{0,1,2\}$. We also have that Assumption (3) holds. We further have that $p(Y=1\,|\,A=a,S=1)=\sum_k p(Y=1\,|\,A=a,U=k,S=1)p(U=k\,|\,A=a,S=1)=p^*(Y=1\,|\,A=a,S=1)$ for $a\in\{0,1\}$. We further have that $p(Y=1\,|\,A=a,U=1,S=s)>p(Y=1\,|\,A=a,U=0,S=s)>p(Y=1\,|\,A=a,U=2,S=s)$, so that $\mathrm{RR}_{UY|a}=p(Y=1\,|\,A=a,U=1,S=s)/p(Y=1\,|\,A=a,U=2,S=s)=\mathrm{RR}^*_{UY|a}$.

E Proof of Theorem 2

In this theorem we show that the sensitivity parameters are restricted by 1 from their definition and that they are variationally independent. The latter is shown by fixing the parameters and then show that there always exists a joint distribution that marginalizes to these parameters, or in other words, that there exists a well-defined joint distribution that gives these sensitivity parameters.

Since

$$
\mathrm{RR}_{UY|S=1} = \max_a \frac{\max_u p(Y=1\,|\,A=a,U=u,S=1)}{\min_u p(Y=1\,|\,A=a,U=u,S=1)},\;\; a\in\{0,1\},
$$

$\mathrm{RR}_{UY|S=1}\geq 1,\ a\in\{0,1\}$, by definition. Define $g(u)=p(U=u\,|\,A=a,S=1)/p(U=u\,|\,A=1-a,S=1)$. Note that $\mathrm{E}[g(U)\,|\,A=1-a,S=1]=\sum_u g(u)p(U=u\,|\,A=1-a,S=1)=\sum_u p(U=u\,|\,A=a,S=1)=1$, which gives $\mathrm{RR}_{AU|a}=\max_u g(u)\geq 1$.

To show the second part of Theorem 2, i.e. that $\{\mathrm{RR}_{UY|S=1},\mathrm{RR}_{AU|1},\mathrm{RR}_{AU|0},p(Y,A\,|\,S=1)\}$ are variation independent, we construct a distribution $p(Y,A,U\,|\,S=1)$ that marginalize to a given set $\{\mathrm{RR}^*_{UY|S=1},\mathrm{RR}^*_{AU|1},\mathrm{RR}^*_{AU|0},p^*(Y,A\,|\,S=1)\}$. We do not consider boundary points in order to avoid technicalities. We construct the distribution as:

1. Let

$$
p(A\,|\,S=1)=p^*(A\,|\,S=1).
$$

2. Let

$$\begin{aligned}
p(U=0\,|\,A=0,S=1) &= \frac{(1-\epsilon)\text{RR}^*_{AU|0}(\text{RR}^*_{AU|1}-1)}{\text{RR}^*_{AU|0}\text{RR}^*_{AU|1}-1}\\
p(U=1\,|\,A=0,S=1) &= \frac{(1-\epsilon)(\text{RR}^*_{AU|0}-1)}{\text{RR}^*_{AU|0}\text{RR}^*_{AU|1}-1}\\
p(U=2\,|\,A=0,S=1) &= \epsilon\\
p(U=0\,|\,A=1,S=1) &= \frac{(1-\epsilon)(\text{RR}^*_{AU|1}-1)}{\text{RR}^*_{AU|0}\text{RR}^*_{AU|1}-1}\\
p(U=1\,|\,A=1,S=1) &= \frac{(1-\epsilon)\text{RR}^*_{AU|1}(\text{RR}^*_{AU|0}-1)}{\text{RR}^*_{AU|0}\text{RR}^*_{AU|1}-1}\\
p(U=2\,|\,A=1,S=1) &= \epsilon.
\end{aligned}$$

We then have that

$$\text{RR}_{AU|1} = \max_u \frac{p(U=u\,|\,A=1,S=1)}{p(U=u\,|\,A=0,S=1)} = \frac{p(U=1\,|\,A=1,S=1)}{p(U=1\,|\,A=0,S=1)} = \text{RR}^*_{AU|1}$$

and that

$$\text{RR}_{AU|0} = \max_u \frac{p(U=u\,|\,A=0,S=1)}{p(U=u\,|\,A=1,S=1)} = \frac{p(U=0\,|\,A=0,S=1)}{p(U=0\,|\,A=1,S=1)} = \text{RR}^*_{AU|0}.$$

3. Let

$$\begin{aligned}
&p(Y=1\,|\,A=0,U=u,S=1) = p^*(Y=1\,|\,A=0,S=1), \quad u\in\{0,1,2\}\\
&p(Y=1\,|\,A=1,U=0,S=1) = p^*(Y=1\,|\,A=1,S=1)\\
&p(Y=1\,|\,A=1,U=1,S=1) = p^*(Y=1\,|\,A=1,S=1)\frac{\text{RR}^*_{UY|S=1}[p(U=1\,|\,A=1,S=1)+\epsilon]}{\text{RR}^*_{UY|S=1}p(U=1\,|\,A=1,S=1)+\epsilon}\\
&p(Y=1\,|\,A=1,U=2,S=1) = p^*(Y=1\,|\,A=1,S=1)\frac{p(U=1\,|\,A=1,S=1)+\epsilon}{\text{RR}^*_{UY|S=1}p(U=1\,|\,A=1,S=1)+\epsilon}.
\end{aligned}$$

Since

$$\begin{aligned}
\frac{\partial}{\partial\epsilon}p(Y=1\,|\,A=1,S=1,U=1) =& p^*(Y=1\,|\,A=1,S=1)\text{RR}^*_{UY|S=1}\\
&\cdot\frac{p(U=1\,|\,A=1,S=1)(\text{RR}^*_{UY|S=1}-1)}{[\text{RR}_{UY|S=1}p(U=1\,|\,A=1,S=1)+\epsilon]^2} > 0
\end{aligned}$$

and

$$\lim_{\epsilon\to 0} p(Y=1\,|\,A=1,U=1,S=1) = p^*(Y=1\,|\,A=1,S=1),$$

we can choose ϵ such that all probabilities are between 0 and 1.

We then get

$$p(Y=1\,|\,A=1,S=1)=\sum_{u} p(Y=1\,|\,A=1,U=u,S=1)p(U=u\,|\,A=1,S=1)$$

$$=p^*(Y=1\,|\,A=1,S=1)p(U=0\,|\,A=1,S=1)$$

$$+\,p^*(Y=1\,|\,A=1,S=1)\frac{\text{RR}^*_{UY|S=1}[p(U=1\,|\,A=1,S=1)+\epsilon]}{\text{RR}^*_{UY|S=1}p(U=1\,|\,A=1,S=1)+\epsilon}p(U=1\,|\,A=1,S=1)$$

$$+\,p^*(Y=1\,|\,A=1,S=1)\frac{p(U=1\,|\,A=1,S=1)+\epsilon}{\text{RR}^*_{UY|S=1}p(U=1\,|\,A=1,S=1)+\epsilon}p(U=2\,|\,A=1,S=1)$$

$$=\frac{p^*(Y=1\,|\,A=1,S=1)}{\text{RR}^*_{UY|S=1}p(U=1\,|\,A=1,S=1)+\epsilon}$$

$$\cdot\Big[\text{RR}^*_{UY|S=1}p(U=0\,|\,A=1,S=1)p(U=1\,|\,A=1,S=1)+\epsilon p(U=0\,|\,A=1,S=1)$$

$$+\,\text{RR}^*_{UY|S=1}p(U=1\,|\,A=1,S=1)^2+\text{RR}^*_{UY|S=1}\epsilon p(U=1\,|\,A=1,S=1)$$

$$+\,p(U=1\,|\,A=1,S=1)p(U=2\,|\,A=1,S=1)+\epsilon p(U=2\,|\,A=1,S=1)]$$

$$=\frac{p^*(Y=1\,|\,A=1,S=1)}{\text{RR}^*_{UY|S=1}p(U=1\,|\,A=1,S=1)+\epsilon}$$

$$\cdot\Big[\text{RR}^*_{UY|S=1}p(U=1\,|\,A=1,S=1)\,\{p(U=0\,|\,A=1,S=1)+p(U=1\,|\,A=1,S=1)+\epsilon\}$$

$$+\,\epsilon\,\{p(U=0\,|\,A=1,S=1)+p(U=1\,|\,A=1,S=1)+p(U=2\,|\,A=1,S=1)\}\,]$$

$$=\frac{p^*(Y=1\,|\,A=1,S=1)}{\text{RR}^*_{UY|S=1}p(U=1\,|\,A=1,S=1)+\epsilon}\Big[\text{RR}^*_{UY|S=1}p(U=1\,|\,A=1,S=1)+\epsilon\Big]$$

$$=p^*(Y=1\,|\,A=1,S=1),$$

since $p(U=2\,|\,A=1,S=1)=\epsilon$, and that

$$p(Y=1\,|\,A=0,S=1)=p^*(Y=1\,|\,A=0,S=1).$$

Since

$$p(Y=1\,|\,A=0,U=1,S=1)>p(Y=1\,|\,A=0,U=0,S=1)>p(Y=1\,|\,A=0,U=2,S=1)$$

we have that

$$\text{RR}_{UY|S=1}=\frac{p(Y=1\,|\,A=0,U=1,S=1)}{p(Y=1\,|\,A=0,U=2,S=1)}=\text{RR}^*_{UY|S=1}.$$

Thus, $\{\text{RR}_{UY|S=1},\ \text{RR}_{AU|1},\ \text{RR}_{AU|0},\ p(Y,A|S=1)\}$ are variation independent.

F Proof of Theorem 3

A bound is sharp if the bias can equal the bound. Similarly, we say that a bound is arbitrarily sharp if the bias can take a value arbitrarily close to the bound. In other words, there exists a distribution and a ξ so

that

$$|Bound - bias| < \xi$$

for $\xi > 0$.

We show that the lower SV bound for the risk ratio in the total population is arbitrarily sharp when $\text{BF}_{00} < 1/p(Y=1 \mid A=0, S=0)$ and $p(S=1 \mid A=a) < \delta_a$, where $0 < \delta_a < 1, a \in \{0,1\}$. The corresponding for the upper bound follows by symmetry. To show that the lower SV bound is arbitrarily sharp we show that it is possible to construct a distribution $p(Y, A, S, U)$ for which Assumption (3) holds, which is consistent with any given set $\{p^*(Y, A \mid S=1), \text{RR}^*_{SU|00}, \text{RR}^*_{SU|11}, \text{RR}^*_{UY|0}, \text{RR}^*_{UY|1}\}$ and is such that CRR_T is arbitrarily close to the bound. We construct the distribution $p(Y, A, S, U)$ in the following steps.

1. We construct $p(A, S)$ as in step 1 of Supplemental Appendix D, but let $p^*_a < \delta_a$.
2. We let U be ternary. We set

$$\begin{aligned}
p(U=0 \mid A=1, S=0) &= 1 - 1/\text{RR}^*_{SU|11} \\
p(U=1 \mid A=1, S=0) &= \epsilon_1/\text{RR}^*_{SU|11} \\
p(U=2 \mid A=1, S=0) &= (1-\epsilon_1)/\text{RR}^*_{SU|11} \\
p(U=0 \mid A=1, S=1) &= 0 \\
p(U=1 \mid A=1, S=1) &= \epsilon_1 \\
p(U=2 \mid A=1, S=1) &= 1-\epsilon_1
\end{aligned}$$

for some $0 < \epsilon_1 < 1$. Furthermore, we set

$$\begin{aligned}
p(U=0 \mid A=0, S=0) &= 0 \\
p(U=1 \mid A=0, S=0) &= \epsilon_0 \\
p(U=2 \mid A=0, S=0) &= 1-\epsilon_0 \\
p(U=0 \mid A=0, S=1) &= 1 - 1/\text{RR}^*_{SU|00} \\
p(U=1 \mid A=0, S=1) &= \epsilon_0/\text{RR}^*_{SU|00} \\
p(U=2 \mid A=0, S=1) &= (1-\epsilon_0)/\text{RR}^*_{SU|00}
\end{aligned}$$

for some $0 < \epsilon_0 < 1$.
We trivially have that $\sum_u p(U=u \mid A=a, S=s) = 1$ for all (a, s) and that $0 \leq p(U=u \mid A=a, S=s) \leq 1$ for all (a, s). We further have that

$$\max_u \frac{p(U=u \mid S=0, A=0)}{p(U=u \mid S=1, A=0)} = \text{RR}^*_{SU|00}$$

and

$$\max_u \frac{p(U=u \mid S=1, A=1)}{p(U=u \mid S=0, A=1)} = \text{RR}^*_{SU|11}.$$

3. Set

$$
\begin{aligned}
p(Y=1\,|\,A=1,U=0,S=s) &= p^*(Y=1\,|\,A=1,S=1)/\text{RR}^*_{UY|1}\\
p(Y=1\,|\,A=1,U=1,S=s) &= p^*(Y=1\,|\,A=1,S=1)\\
p(Y=1\,|\,A=1,U=2,S=s) &= p^*(Y=1\,|\,A=1,S=1)\\
p(Y=1\,|\,A=0,U=0,S=s) &= p^*(Y=1\,|\,A=0,S=1)\text{BF}^*_{00}/\text{RR}^*_{UY|0}\\
p(Y=1\,|\,A=0,U=1,S=s) &= p^*(Y=1\,|\,A=0,S=1)\text{BF}^*_{00}\\
p(Y=1\,|\,A=0,U=2,S=s) &= p^*(Y=1\,|\,A=0,S=1)\text{BF}^*_{00}
\end{aligned}
$$

We have that $0 \leq p(Y=1\,|\,A=a,U=u,S=s) \leq 1$ provided that $\text{BF}^*_{00} < 1/p^*(Y=1\,|\,A=0,S=1)$. We also have that Assumption (3) holds. We further have that

$$\frac{\max_u p(Y=1\,|\,U=u,A=0)}{\min_u p(Y=1\,|\,U=u,A=0)} = \text{RR}^*_{UY|0}$$

and

$$\frac{\max_u p(Y=1\,|\,U=u,A=1)}{\min_u p(Y=1\,|\,U=u,A=1)} = \text{RR}^*_{UY|1}.$$

We finally have that

$$
\begin{aligned}
&p(Y=1\,|\,A=1,S=1)\\
&\quad=\sum_u p(Y=1\,|\,A=1,U=u,S=1)p(U=u\,|\,A=1,S=1)\\
&\quad=p^*(Y=1\,|\,A=1,S=1).
\end{aligned}
$$

$$
\begin{aligned}
&p(Y=1\,|\,A=0,S=1)\\
&\quad=\sum_u p(Y=1\,|\,A=0,U=u,S=1)p(U=u\,|\,A=0,S=1)\\
&\quad=p^*(Y=1\,|\,A=0,S=1),
\end{aligned}
$$

$$
\begin{aligned}
&p(Y=1\,|\,A=1,S=0)\\
&\quad=\sum_u p(Y=1\,|\,A=1,U=u,S=0)p(U=u\,|\,A=1,S=0)\\
&\quad=p^*(Y=1\,|\,A=1,S=1)/\text{BF}^*_{11}
\end{aligned}
$$

and

$$
\begin{aligned}
&p(Y=1\,|\,A=0,S=0)\\
&\quad=\sum_u p(Y=1\,|\,A=0,U=u,S=0)p(U=u\,|\,A=0,S=0)\\
&\quad=\text{BF}^*_{00}p^*(Y=1\,|\,A=0,S=1),
\end{aligned}
$$

provided that $\mathrm{BF}^*_{00} < 1/p^*(Y = 1 \mid A = 0, S = 1)$, so that

$$\begin{aligned}\frac{p(Y=1\mid A=1)}{p(Y=1\mid A=0)} &= \frac{\sum_s p(Y=1\mid A=1,S=s)p(S=s\mid A=1)}{\sum_s p(Y=1\mid A=0,S=s)p(S=s\mid A=0)}\\ &= \frac{\begin{array}{r}p^*(Y=1\mid A=1,S=1)/\mathrm{BF}^*_{11}p(S=0\mid A=1)\\ +\,p^*(Y=1\mid A=1,S=1)p(S=1\mid A=1)\end{array}}{\begin{array}{r}p^*(Y=1\mid A=0,S=1)\mathrm{BF}^*_{00}p(S=0\mid A=0)\\ +\,p^*(Y=1\mid A=0,S=1)p(S=1\mid A=0)\end{array}}\\ &> \frac{\begin{array}{r}p^*(Y=1\mid A=1,S=1)/\mathrm{BF}^*_{11}(1-\delta_1)\\ +\,p^*(Y=1\mid A=1,S=1)\delta_1\end{array}}{\begin{array}{r}p^*(Y=1\mid A=0,S=1)\mathrm{BF}^*_{00}(1-\delta_0)\\ +\,p^*(Y=1\mid A=0,S=1)\delta_0\end{array}}\end{aligned}$$

where the right hand side is arbitrarily close to $\frac{p^*(Y=1\mid A=1,S=1)}{p^*(Y=1\mid A=0,S=1)\mathrm{BF}^*_{00}\mathrm{BF}^*_{11}}$ for small δ_a, $a \in \{0, 1\}$. In other words, under the specified conditions and for small δ_a, the bias can arbitrarily close to the SV bound, and this bound is thus arbitrarily sharp.

G Proof of Theorem 4

To show that the lower bound for the risk ratio in the subpopulation is sharp when $\mathrm{BF}_1 \leq 1/p(Y = 1 \mid A = 0, S = 1)$, we construct a distribution that marginalizes to any given set $\{\mathrm{RR}^*_{AU|1}, \mathrm{RR}^*_{UY|S=1}, p^*(Y, A, U \mid S = 1)\}$ such that $\mathrm{BF}^*_1 \leq 1/p^*(Y = 1 \mid A = 0, S = 1)$ and $\mathrm{CRR}_S = \mathrm{RR}^*_S/\mathrm{BF}^*_1$.

1. Let

$$p(A \mid S = 1) = p^*(A \mid S = 1).$$

2. Let

$$\begin{aligned}p(U=1\mid A=1,S=1) &= 1\\ p(U=1\mid A=0,S=1) &= \frac{1}{\mathrm{RR}^*_{AU|1}}.\end{aligned}$$

Then $\mathrm{RR}_{AU|1} = \mathrm{RR}^*_{AU|1}$.

3. Let

$$\begin{aligned}p(Y=1\mid A=0,U=0,S=1) &= p^*(Y=1\mid A=0,S=1)\cdot \mathrm{BF}^*_1/\mathrm{RR}^*_{UY|S=1}\\ p(Y=1\mid A=0,U=1,S=1) &= p^*(Y=1\mid A=0,S=1)\cdot \mathrm{BF}^*_1\\ p(Y=1\mid A=1,U=0,S=1) &= p^*(Y=1\mid A=1,S=1)/\mathrm{RR}^*_{UY|S=1}\\ p(Y=1\mid A=1,U=1,S=1) &= p^*(Y=1\mid A=1,S=1).\end{aligned}$$

Provided that

$$\mathrm{BF}^*_1 \leq \frac{1}{p^*(Y=1\mid A=0,S=1)},$$

$\text{RR}_{UY|S=1} = \text{RR}^*_{UY|S=1}$. Furthermore,

$$\begin{aligned}
p(U=0 \mid S=1) &= p(U=0 \mid A=0, S=1)p(A=0 \mid S=1) \\
&\quad + p(U=0 \mid A=1, S=1)p(A=1 \mid S=1) \\
&= \left(1 - \frac{1}{\text{RR}^*_{AU|1}}\right) p(A=0 \mid S=1),
\end{aligned}$$

$$\begin{aligned}
p(U=1 \mid S=1) &= p(U=1 \mid A=0, S=1)p(A=0 \mid S=1) \\
&\quad + p(U=1 \mid A=1, S=1)p(A=1 \mid S=1) \\
&= \frac{1}{\text{RR}^*_{AU|1}} p(A=0 \mid S=1) + p(A=1 \mid S=1),
\end{aligned}$$

$$\begin{aligned}
&p(Y^1=1 \mid S=1) = p(Y=1 \mid A=1, U=0, S=1)p(U=0 \mid S=1) \\
&+ p(Y=1 \mid A=1, U=1, S=1)p(U=1 \mid S=1) \\
&= \frac{p^*(Y=1 \mid A=1, S=1)}{\text{RR}^*_{UY|S=1}} \cdot \left(1 - \frac{1}{\text{RR}^*_{AU|1}}\right) \cdot p(A=0 \mid S=1) \\
&+ p^*(Y=1 \mid A=1, S=1) \cdot \left(p(A=1 \mid S=1) + p(A=0 \mid S=1) \cdot \frac{1}{\text{RR}^*_{AU|1}}\right) \\
&= p^*(Y=1 \mid A=1, S=1) \cdot [p(A=1 \mid S=1) + p(A=0 \mid S=1) \\
&\cdot \left(\frac{1}{\text{RR}^*_{UY|S=1}} - \frac{1}{\text{RR}^*_{AU|1}\text{RR}^*_{UY|S=1}} + \frac{1}{\text{RR}^*_{AU|1}}\right)\Bigg] \\
&= p^*(Y=1 \mid A=1, S=1) \cdot \left(p(A=1 \mid S=1) + p(A=0 \mid S=1) \cdot \frac{1}{\text{BF}^*_1}\right)
\end{aligned}$$

and

$$\begin{aligned}
&p(Y^0=1 \mid S=1) = p(Y=1 \mid A=0, U=0, S=1)p(U=0 \mid S=1) \\
&+ p(Y=1 \mid A=0, U=1, S=1)p(U=1 \mid S=1) \\
&= \frac{p^*(Y=1 \mid A=0, S=1) \cdot \text{BF}^*_1}{\text{RR}^*_{UY|S=1}} \cdot \left(1 - \frac{1}{\text{RR}^*_{AU|S=1}}\right) \cdot p(A=0 \mid S=1) \\
&+ p^*(Y=1 \mid A=0, S=1) \cdot \text{BF}^*_1 \cdot \left(p(A=1 \mid S=1) + p(A=0 \mid S=1) \cdot \frac{1}{\text{RR}^*_{AU|1}}\right) \\
&= p^*(Y=1 \mid A=0, S=1) \cdot \text{BF}^*_1 \cdot [p(A=1 \mid S=1) + p(A=0 \mid S=1) \\
&\cdot \left(\frac{1}{\text{RR}^*_{UY|S=1}} - \frac{1}{\text{RR}^*_{AU|1}\text{RR}^*_{UY|S=1}} + \frac{1}{\text{RR}^*_{AU|1}}\right)\Bigg] \\
&= p^*(Y=1 \mid A=0, S=1) \cdot \text{BF}^*_1 \cdot \left(p(A=1 \mid S=1) + p(A=0 \mid S=1) \cdot \frac{1}{\text{BF}^*_1}\right).
\end{aligned}$$

Thus, we get

$$\text{CRR}_S = \frac{p^*(Y=1 \mid A=1, S=1)}{p^*(Y=1 \mid A=0, S=1)\text{BF}_1^*}.$$

Lastly, we have

$$\begin{aligned} p(Y=1 \mid A=1, S=1) &= p(Y=1 \mid A=1, U=0, S=1)p(U=0 \mid A=1, S=1) \\ &\quad + p(Y=1 \mid A=1, U=1, S=1)p(U=1 \mid A=1, S=1) \\ &= p^*(Y=1 \mid A=1, S=1) \end{aligned}$$

and

$$\begin{aligned} p(Y=1 \mid A=0, S=1) &= p(Y=1 \mid A=0, U=0, S=1)p(U=0 \mid A=0, S=1) \\ &\quad + p(Y=1 \mid A=0, U=1, S=1)p(U=1 \mid A=0, S=1) \\ &= \frac{p^*(Y=1 \mid A=0, S=1)\text{BF}_1^*}{\text{RR}^*_{UY\mid S=1}} \cdot \left(1 - \frac{1}{\text{RR}^*_{AU\mid 1}}\right) \\ &\quad + p^*(Y=1 \mid A=0, S=1)\text{BF}_1^* \cdot \frac{1}{\text{RR}^*_{AU\mid 1}} \\ &= p^*(Y=1 \mid A=0, S=1)\text{BF}_1^* \cdot \frac{1}{\text{BF}_1^*} \\ &= p^*(Y=1 \mid A=0, S=1). \end{aligned}$$

Thus, the lower bound for the risk ratio in the subpopulation is sharp.

To show that the upper bound for the risk ratio in the subpopulation are sharp when $\text{BF}_0 \leq 1/p(Y=1 \mid A=1, S=1)$, we construct a distribution that marginalizes to any given set $\{\text{RR}^*_{AU\mid 0}, \text{RR}^*_{UY\mid S=1}, p^*(Y, A, U \mid S=1)\}$ such that $\text{BF}_0^* \leq 1/p^*(Y=1 \mid A=1, S=1)$ and $\text{CRR}_S = \text{RR}^*_S\text{BF}^*_0$.

1. Let

$$p(A \mid S=1) = p^*(A \mid S=1).$$

2. Let

$$\begin{aligned} p(U=1 \mid A=0, S=1) &= 1 \\ p(U=1 \mid A=1, S=1) &= \frac{1}{\text{RR}^*_{AU\mid 0}}. \end{aligned}$$

Then $\text{RR}_{AU\mid 0} = \text{RR}^*_{AU\mid 0}$.

3. Let

$$\begin{aligned} p(Y=1 \mid A=0, U=0, S=1) &= p^*(Y=1 \mid A=0, S=1)/\text{RR}^*_{UY\mid S=1} \\ p(Y=1 \mid A=0, U=1, S=1) &= p^*(Y=1 \mid A=0, S=1) \\ p(Y=1 \mid A=1, U=0, S=1) &= p^*(Y=1 \mid A=1, S=1)\text{BF}^*_0/\text{RR}^*_{UY\mid S=1} \\ p(Y=1 \mid A=1, U=1, S=1) &= p^*(Y=1 \mid A=1, S=1)\text{BF}^*_0. \end{aligned}$$

Provided that

$$\mathrm{BF}_0^* \leq \frac{1}{p^*(Y=1 \mid A=1, S=1)},$$

$\mathrm{RR}_{UY|S=1} = \mathrm{RR}^*_{UY|S=1}$. We have that

$$\mathrm{CRR}_S = \frac{p^*(Y=1 \mid A=1, S=1)\mathrm{BF}_0^*}{p^*(Y=1 \mid A=0, S=1)}.$$

and

$$\begin{aligned} p(Y=1 \mid A=0, S=1) &= p(Y=1 \mid A=0, U=0, S=1)p(U=0 \mid A=0, S=1) \\ &\quad + p(Y=1 \mid A=0, U=1, S=1)p(U=1 \mid A=0, S=1) \\ &= p^*(Y=1 \mid A=0, S=1) \end{aligned}$$

and

$$\begin{aligned} p(Y=1 \mid A=1, S=1) &= p(Y=1 \mid A=1, U=0, S=1)p(U=0 \mid A=1, S=1) \\ &\quad + p(Y=1 \mid A=1, U=1, S=1)p(U=1 \mid A=1, S=1) \\ &= \frac{p^*(Y=1 \mid A=1, S=1)\mathrm{BF}_0^*}{\mathrm{RR}^*_{UY|S=1}} \cdot \left(1 - \frac{1}{\mathrm{RR}^*_{AU|0}}\right) \\ &\quad + p^*(Y=1 \mid A=1, S=1)\mathrm{BF}_0^* \cdot \frac{1}{\mathrm{RR}^*_{AU|0}} \\ &= p^*(Y=1 \mid A=1, S=1)\mathrm{BF}_0^* \cdot \frac{1}{\mathrm{BF}_0^*} \\ &= p^*(Y=1 \mid A=1, S=1). \end{aligned}$$

Thus, the upper bound for the risk ratio in the subpopulation is sharp.

The lower bound is not sharp if $\mathrm{BF}_1 > 1/p(Y=1 \mid A=0, S=1)$. Following the notation in Ding and VanderWeele (2016), we have that

$$\mathrm{CRR}_S^{-1} = w \cdot \mathrm{CRR}_+^{-1} + \mathrm{CRR}_-^{-1}.$$

Furthermore, we have that

$$\mathrm{CRR}_+ = \frac{E[p(Y=1 \mid A=0, S=1) \mid A=1, S=1]}{p(Y=1 \mid A=0, S=1)}$$

and

$$\mathrm{CRR}_- = \frac{p(Y=1 \mid A=1, S=1)}{E[p(Y=1 \mid A=1, S=1) \mid A=0, S=1]}.$$

Ding and VanderWeele (2016) show that $\mathrm{CRR}_+ \leq \mathrm{BF}_1$ and $\mathrm{CRR}_- \leq \mathrm{BF}_1$ which gives the bound. However, $E[p(Y=1 \mid A=0, S=1) \mid A=1, S=1] \leq 1$ which gives the inequality $\mathrm{CRR}_+ \leq$

$\min[\mathrm{BF}_1, 1/p(Y=1\,|\,A=0,S=1)]$. Thus, if $\mathrm{BF}_1 > 1/p(Y=1\,|\,A=0,S=1)$ we get

$$\begin{aligned}\mathrm{CRR}_S^{-1} &= w\cdot \mathrm{CRR}_+^{-1} + \mathrm{CRR}_-^{-1}\\ &\geq w\cdot p(Y=1\,|\,A=0,S=1) + (1-w)\cdot \mathrm{BF}_1^{-1}\\ &> w\cdot \mathrm{BF}_1^{-1} + (1-w)\cdot \mathrm{BF}_1^{-1}\\ &= \mathrm{BF}_1^{-1}\end{aligned}$$

which results in $\mathrm{CRR}_S < \mathrm{BF}_1$, i.e. the lower bound is not sharp. Similar arguments can be used to show that the upper bound is not sharp if $\mathrm{BF}_0 > 1/p(Y=1\,|\,A=1,S=1)$.

H Proof of Theorem 5

To show that the lower SV bound for the risk difference in the subpopulation is arbitrarily sharp when $\mathrm{BF}_1 \leq 1/p(Y=1\,|\,A=0,S=1)$ and $p(A=1\,|\,S=1) < \delta_1$ and $p(Y=1\,|\,A=1,S=1)\cdot(1-1/\mathrm{BF}_1) < p(Y=1\,|\,A=0,S=1)\cdot(\mathrm{BF}_1-1)$ or when $p(A=0\,|\,S=1) < \delta_0$ and $p(Y=1\,|\,A=1,S=1)\cdot(1-1/\mathrm{BF}_1) > p(Y=1\,|\,A=0,S=1)\cdot(\mathrm{BF}_1-1)$, and that the alternative lower bound in (**??**) is sharp when $\mathrm{BF}_1 \leq 1/p(Y=1\,|\,A=0,S=1)$, we use the same distribution as for the risk ratio in the subpopulation. Furthermore, from the eAppendix 2e in Smith and VanderWeele (2019), we have that

$$\begin{aligned}\mathrm{CRD}_S^{+} &= p(Y=1\,|\,A=1,S=1) - \sum_u p(Y=1\,|\,A=0,U=u,S=1)p(U=u\,|\,A=1,S=1)\\ &= p(Y=1\,|\,A=1,S=1) - \frac{p^*(Y=1\,|\,A=0,S=1)\mathrm{RR}^*_{U1}}{\mathrm{RR}^*_{UY|S=1}}\cdot 0\\ &\quad - p^*(Y=1\,|\,A=0,S=1)\mathrm{BF}_1^*\cdot 1\\ &= p(Y=1\,|\,A=1,S=1) - p^*(Y=1\,|\,A=0,S=1)\mathrm{BF}_1^*\\ &= p(Y=1\,|\,A=1,S=1) - p(Y=1\,|\,A=0,S=1)\mathrm{BF}_1\end{aligned}$$

and

$$\begin{aligned}\mathrm{CRD}_S^{-} &= \sum_u p(Y=1\,|\,A=1,U=u,S=1)p(U=u\,|\,A=0,S=1) - p(Y=1\,|\,A=0,S=1)\\ &= \frac{p^*(Y=1\,|\,A=1,S=1)}{\mathrm{RR}^*_{UY|S=1}}\cdot\left(1-\frac{1}{\mathrm{RR}_{AU|1}}\right)\\ &\quad + p^*(Y=1\,|\,A=1,S=1)\cdot\frac{1}{\mathrm{RR}^*_{AU|1}} - p(Y=1\,|\,A=0,S=1)\\ &= \frac{p^*(Y=1\,|\,A=1,S=1)}{\mathrm{BF}_1^*} - p(Y=1\,|\,A=0,S=1)\\ &= \frac{p(Y=1\,|\,A=1,S=1)}{\mathrm{BF}_1} - p(Y=1\,|\,A=0,S=1).\end{aligned}$$

We thus get

$$\mathrm{RD}_S - \mathrm{CRD}_S^{+} = p(Y=1\,|\,A=0,S=1)\cdot(\mathrm{BF}_1 - 1)$$

and

$$\mathrm{RD}_S - \mathrm{CRD}_S^- = p(Y=1 \mid A=1, S=1) \cdot (1 - 1/\mathrm{BF}_1).$$

Since $\mathrm{CRD}_S = p(A=1 \mid S=1)\mathrm{CRD}_S^+ + p(A=0 \mid S=1)\mathrm{CRD}_S^-$, we get that

$$\begin{aligned}\mathrm{RD}_S - \mathrm{CRD}_S = {} & p(A=0 \mid S=1) \cdot p(Y=1 \mid A=1, S=1) \cdot (1 - 1/\mathrm{BF}_1) \\ & + p(A=1 \mid S=1) \cdot p(Y=1 \mid A=0, S=1) \cdot (\mathrm{BF}_1 - 1),\end{aligned}$$

which is equal to the lower bound in (**??**), i.e. the lower bound is sharp. Furthermore, if $p(A = 1 \mid S=1) < \delta_1$ and $p(Y=1 \mid A=1, S=1) \cdot (1 - 1/\mathrm{BF}_1) < p(Y=1 \mid A=0, S=1) \cdot (\mathrm{BF}_1 - 1)$ or if $p(A=0 \mid S=1) < \delta_0$ and $p(Y=1 \mid A=1, S=1) \cdot (1 - 1/\mathrm{BF}_1) > p(Y=1 \mid A=0, S=1) \cdot (\mathrm{BF}_1 - 1)$, the lower SV bound is arbitrarily sharp.

The lower bound is not sharp if $\mathrm{BF}_1 > 1/p(Y=1 \mid A=0, S=1)$. We have already shown that $\mathrm{CRR}_S < \mathrm{BF}_1$ in the proof for the risk ratio. Since this is a part for the bound for the risk difference as well, the same conclusions hold.

The proofs for the upper SV bound and alternative upper bound in (**??**) are done by following the same procedure.

I Proof of Theorem 6

I.1 Proof of validness

We have that

$$\begin{aligned} & p(Y=1 \mid A=a) \\ & = \sum_{s \in \{0,1\}} p(Y=1 \mid A=a, S=s) p(S=s \mid A=a) \\ & = p(Y=1 \mid A=a, S=1) p(S=1 \mid A=a) \\ & + E\{p(Y=1 \mid A=a, S=0, U) \mid A=a, S=0\} p(S=0 \mid A=a) \\ & = p(Y=1 \mid A=a, S=1) p(S=1 \mid A=a) \\ & + E\{p(Y=1 \mid A=a, S=1, U) \mid A=a, S=0\} p(S=0 \mid A=a) \\ & = p(Y=1 \mid A=a, S=1)[p(S=1 \mid A=a) + \mathrm{CRR}_a p(S=0 \mid A=a)], \end{aligned}$$

where the first and second equalities follow from the law of total probability, the third from Assumption (3), and in the fourth equality we have defined

$$\mathrm{CRR}_a = \frac{E\{p(Y=1 \mid A=a, S=1, U) \mid A=a, S=0\}}{p(Y=1 \mid A=a, S=1)}.$$

It follows from Lemma A.3 in the supplementary material of Ding and Vanderweele (2016) that

$$1/\mathrm{BF}_{a1} \le \mathrm{CRR}_a \le \mathrm{BF}_{a0}.$$

However, we also have that

$$E\{p(Y=1 \mid A=a, S=1, U) \mid A=a, S=0\} \le 1,$$

so that

$$1/\mathrm{BF}_{a1} \leq \mathrm{CRR}_a \leq \min\{\mathrm{BF}_{a0}, 1/p(Y=1 \mid A=a, S=1)\},$$

which gives the bounds in (6).

I.2 Proof of sharpness

We show that the lower bound for $p(Y=1 \mid A=1)$ and the upper bound for $p(Y=1 \mid A=0)$ are simultaneously sharp. The converse follows by symmetry. To show that the lower bound for $p(Y=1 \mid A=1)$ and the upper bound for $p(Y=1 \mid A=0)$ are simultaneously sharp we show that it is possible to construct a distribution $p(Y,A,S,U)$ for which Assumption (3) holds, which is consistent with any given set given set $\{p^*(Y,A \mid S=1), p^*(S=1 \mid A=1), p^*(S=1 \mid A=0), \mathrm{RR}^*_{SU|00}, \mathrm{RR}^*_{SU|11}, \mathrm{RR}^*_{UY|0}, \mathrm{RR}^*_{UY|1}\}$ and is such that $p(Y=1 \mid A=1) = l_1$ and $p(Y=1 \mid A=0) = u_0$. We construct the distribution $p(Y,A,S,U)$ in the following steps.

1. We construct $p(A,S)$ as in step 1 of Supplemental Appendix D.
2. We let U be ternary. We set

$$\begin{aligned}
p(U=0 \mid A=1, S=0) &= 1 - 1/\mathrm{RR}^*_{SU|11} \\
p(U=1 \mid A=1, S=0) &= \epsilon_1/\mathrm{RR}^*_{SU|11} \\
p(U=2 \mid A=1, S=0) &= (1-\epsilon_1)/\mathrm{RR}^*_{SU|11} \\
p(U=0 \mid A=1, S=1) &= 0 \\
p(U=1 \mid A=1, S=1) &= \epsilon_1 \\
p(U=2 \mid A=1, S=1) &= 1-\epsilon_1
\end{aligned}$$

for some $0 < \epsilon_1 < 1$. If $\mathrm{BF}^*_{00} < 1/p^*(Y=1 \mid A=0, S=1)$, then we set

$$\begin{aligned}
p(U=0 \mid A=0, S=0) &= 0 \\
p(U=1 \mid A=0, S=0) &= \epsilon_0 \\
p(U=2 \mid A=0, S=0) &= 1-\epsilon_0 \\
p(U=0 \mid A=0, S=1) &= 1 - 1/\mathrm{RR}^*_{SU|00} \\
p(U=1 \mid A=0, S=1) &= \epsilon_0/\mathrm{RR}^*_{SU|00} \\
p(U=2 \mid A=0, S=1) &= (1-\epsilon_0)/\mathrm{RR}^*_{SU|00}
\end{aligned}$$

for some $0 < \epsilon_0 < 1$. If $\text{BF}^*_{00} \geq 1/p^*(Y=1 \mid A=0, S=1)$, then we set

$$\begin{aligned}
p(U=0 \mid A=0, S=0) &= 0 \\
p(U=1 \mid A=0, S=0) &= 0 \\
p(U=2 \mid A=0, S=0) &= 1 \\
p(U=0 \mid A=0, S=1) &= 1 - \frac{\{p(Y=1 \mid A=0, S=1) - 1/\text{BF}^*_{00}\}\text{RR}^*_{UY|0}}{\text{RR}^*_{UY|0} - 1} - 1/\text{RR}^*_{SU|00} \\
p(U=1 \mid A=0, S=1) &= \frac{\{p(Y=1 \mid A=0, S=1) - 1/\text{BF}^*_{00}\}\text{RR}^*_{UY|0}}{\text{RR}^*_{UY|0} - 1} \\
p(U=2 \mid A=0, S=1) &= 1/\text{RR}^*_{SU|00}
\end{aligned}$$

We trivially have that $\sum_u p(U=u \mid A=a, S=s) = 1$ for all (a,s). We also have that $0 \leq p(U=u \mid A=a, S=s) \leq 1$ for all (a,s). In particular, we have that

$$\begin{aligned}
\frac{\{p(Y=1 \mid A=0, S=1) - 1/\text{BF}^*_{00}\}\text{RR}^*_{UY|0}}{\text{RR}^*_{UY|0} - 1} &\leq 1 - 1/\text{RR}^*_{SU|00} \\
&\Leftrightarrow \\
p(Y=1 \mid A=0, S=1)\text{RR}^*_{UY|0} - \frac{\text{RR}^*_{SU|00} + \text{RR}^*_{UY|0} - 1}{\text{RR}^*_{SU|00}} &\leq (1 - 1/\text{RR}^*_{SU|00})(\text{RR}^*_{UY|0} - 1) \\
&\Leftrightarrow \\
p(Y=1 \mid A=0, S=1)\text{RR}^*_{UY|0}\text{RR}^*_{SU|00} &\leq \text{RR}^*_{UY|0}\text{RR}^*_{SU|00},
\end{aligned}$$

which is true. This implies that $p(U=1 \mid A=0, S=1) \leq 1$ and $p(U=2 \mid A=0, S=1) \leq 1$. We further have that

$$\max_u \frac{p(U=u \mid S=0, A=0)}{p(U=u \mid S=1, A=0)} = \text{RR}^*_{SU|00}$$

and

$$\max_u \frac{p(U=u \mid S=1, A=1)}{p(U=u \mid S=0, A=1)} = \text{RR}^*_{SU|11}.$$

3. Set

$$\begin{aligned}
p(Y=1 \mid A=1, U=0, S=s) &= p^*(Y=1 \mid A=1, S=1)/\text{RR}^*_{UY|1} \\
p(Y=1 \mid A=1, U=1, S=s) &= p^*(Y=1 \mid A=1, S=1) \\
p(Y=1 \mid A=1, U=2, S=s) &= p^*(Y=1 \mid A=1, S=1)
\end{aligned}$$

If $\text{BF}^*_{00} < 1/p^*(Y=1 \mid A=0, S=1)$, then we set

$$\begin{aligned}
p(Y=1 \mid A=0, U=0, S=s) &= p^*(Y=1 \mid A=0, S=1)\text{BF}^*_{00}/\text{RR}^*_{UY|0} \\
p(Y=1 \mid A=0, U=1, S=s) &= p^*(Y=1 \mid A=0, S=1)\text{BF}^*_{00} \\
p(Y=1 \mid A=0, U=2, S=s) &= p^*(Y=1 \mid A=0, S=1)\text{BF}^*_{00}
\end{aligned}$$

If $\text{BF}_{00}^* \geq 1/p^*(Y=1 \mid A=0, S=1)$, then we set

$$\begin{aligned} p(Y=1 \mid A=0, U=0, S=s) &= 1/\text{RR}_{UY|0}^* \\ p(Y=1 \mid A=0, U=1, S=s) &= 1 \\ p(Y=1 \mid A=0, U=2, S=s) &= 1 \end{aligned}$$

We have that $0 \leq p(Y=1 \mid A=a, U=u, S=s) \leq 1$. We also have that Assumption (3) holds. We further have that

$$\frac{\max_u p(Y=1 \mid U=u, A=0)}{\min_u p(Y=1 \mid U=u, A=0)} = \text{RR}_{UY|0}^*$$

and

$$\frac{\max_u p(Y=1 \mid U=u, A=1)}{\min_u p(Y=1 \mid U=u, A=1)} = \text{RR}_{UY|1}^*.$$

We finally have that

$$\begin{aligned} & p(Y=1 \mid A=1, S=1) \\ & \quad = \sum_u p(Y=1 \mid A=1, U=u, S=1) p(U=u \mid A=1, S=1) \\ & \quad = p^*(Y=1 \mid A=1, S=1). \end{aligned}$$

$$\begin{aligned} & p(Y=1 \mid A=0, S=1) \\ & \quad = \sum_u p(Y=1 \mid A=0, U=u, S=1) p(U=u \mid A=0, S=1) \\ & \quad = p^*(Y=1 \mid A=0, S=1), \end{aligned}$$

$$\begin{aligned} & p(Y=1 \mid A=1, S=0) \\ & \quad = \sum_u p(Y=1 \mid A=1, U=u, S=0) p(U=u \mid A=1, S=0) \\ & \quad = p^*(Y=1 \mid A=1, S=1)/\text{BF}_{11}^* \end{aligned}$$

and

$$\begin{aligned} & p(Y=1 \mid A=0, S=0) \\ & \quad = \sum_u p(Y=1 \mid A=0, U=u, S=0) p(U=u \mid A=0, S=0) \\ & \quad = \begin{cases} \text{BF}_{00}^* p^*(Y=1 \mid A=0, S=1) & \text{if} \quad \text{BF}_{00}^* < 1/p^*(Y=1 \mid A=0, S=1) \\ 1 & \text{if} \quad \text{BF}_{00}^* \geq 1/p^*(Y=1 \mid A=0, S=1) \end{cases} \end{aligned}$$

so that $p(Y=1 \mid A=1) = l_1$ and $p(Y=1 \mid A=0) = u_0$.

J Proof of Theorem 7

J.1 Proof of validness

From the conditional independence assumption in (??), and the law of total probability we have that

$$\begin{aligned}
&p(Y^a = 1 \mid S = 1)\\
&= E\{p(Y = 1 \mid A = a, S = 1, U)\}\\
&= \sum_{a' \in \{0,1\}} E\{p(Y = 1 \mid A = a, S = 1, U) \mid A = a', S = 1\}p(A = a \mid S = 1)\\
&= p(Y = 1 \mid A = a, S = 1)p(A = a \mid S = 1)\\
&+ E\{p(Y = 1 \mid A = a, S = 1, U) \mid A = 1 - a, S = 1\}p(A = 1 - a \mid S = 1)\\
&= p(Y = 1 \mid A = a, S = 1)[p(A = a \mid S = 1) + \text{CRR}_{AaY}\, p(A = 1 - a \mid S = 1)].
\end{aligned}$$

The fourth equality comes from the definition

$$\text{CRR}_{AaY} = \frac{E\{p(Y = 1 \mid A = a, S = 1, U) \mid A = 1 - a, S = 1\}}{p(Y = 1 \mid A = a, S = 1)}.$$

It follows from Lemma A.3 in the supplementary material of Ding and Vanderweele (2016) that

$$1/\text{BF}_a \le \text{CRR}_{AaY} \le \text{BF}_{(1-a)}.$$

However, we also have that

$$E\{p(Y = 1 \mid A = a, S = 1, U) \mid A = 1 - a, S = 1\} \le 1,$$

so that

$$1/\text{BF}_a \le \text{CRR}_{AaY} \le \min\{\text{BF}_{(1-a)}, 1/p(Y = 1 \mid A = a, S = 1)\},$$

which gives the bounds in (9).

J.2 Proof of sharpness

We show that the lower bound for $p(Y^1 = 1 \mid S = 1)$ and the upper bound for $p(Y^0 = 1 \mid S = 1)$ are simultaneously sharp. The converse follows by symmetry. To show that the lower bound for $p(Y^1 = 1 \mid S = 1)$ and the upper bound for $p(Y^0 = 1 \mid S = 1)$ are simultaneously sharp we show that it is possible to construct a distribution $p(Y, A, U \mid S = 1)$ for which Assumption (4) holds, which is consistent with any given set $\{p^*(Y, A \mid S = 1), \text{RR}^*_{AU|1}, \text{RR}^*_{UY|S=1}\}$ and is such that $p(Y^1 = 1 \mid S = 1) = l_1$ and $p(Y^0 = 1 \mid S = 1) = u_0$. We construct the distribution $p(Y, A, U \mid S = 1)$ in the following steps.

1. Let $p(A \mid S = 1) = p^*(A \mid S = 1)$
2. We let U be binary. We set

$$\begin{aligned}
p(U = 1 \mid A = 1, S = 1) &= 1\\
p(U = 1 \mid A = 0, S = 1) &= 1/x
\end{aligned}$$

where x is an arbitrary number such that $x \ge \text{BF}^*_1$. We have that $0 < p(U = 1 \mid A = a, S = 1) \le 1$ for $a \in \{0, 1\}$, and that $\text{RR}_{AU|1} = p(U = 1 \mid A = 1, S = 1)/p(U = 1 \mid A = 0, S = 1) = x$.

3. Set

$$\begin{aligned} p(Y=1\mid A=0,U=0,S=1) &= \frac{p^*(Y=1\mid A=0,S=1)-1/x}{1-1/x} \\ p(Y=1\mid A=0,U=1,S=1) &= 1 \\ p(Y=1\mid A=1,U=0,S=1) &= p^*(Y=1\mid A=1,S=1)\frac{1/\mathrm{BF}_1^*-1/x}{1-1/x} \\ p(Y=1\mid A=1,U=1,S=1) &= p^*(Y=1\mid A=1,S=1) \end{aligned}$$

We have that $0 \le p(Y=1\mid A=a,U=u,S=1) \le 1$ for $(a,u) \in \{0,1\}$. We further have that

$$\begin{aligned} & p(Y=1\mid A=0,S=1) \\ &= \sum_u p(Y=1\mid A=0,U=u,S=1)p(U=u\mid A=0,S=1) \\ &= 1\times 1/x + \frac{p^*(Y=1\mid A=0,S=1)-1/x}{1-1/x}\times(1-1/x) \\ &= p^*(Y=1\mid A=0,S=1) \end{aligned}$$

and

$$\begin{aligned} & p(Y=1\mid A=1,S=1) \\ &= \sum_u p(Y=1\mid A=1,U=u,S=1)p(U=u\mid A=1,S=1) \\ &= p^*(Y=1\mid A=1,S=1)\times 1 + p^*(Y=1\mid A=0,S=1)\times\frac{1/\mathrm{BF}_1^*-1/x}{1-1/x}\times 0 \\ &= p^*(Y=1\mid A=1,S=1) \end{aligned}$$

We further have that

$$\begin{aligned} & \frac{p(Y=1\mid A=1,U=1,S=1)}{p(Y=1\mid A=1,U=0,S=1)} = \frac{1-1/x}{1/\mathrm{BF}_1^*-1/x} \\ & > \frac{p(Y=1\mid A=0,U=1,S=1)}{p(Y=1\mid A=0,U=0,S=1)} = \frac{1-1/x}{p^*(Y=1\mid A=0,S=1)-1/x} \\ & \ge 1. \end{aligned}$$

so that

$$\begin{aligned} \mathrm{RR}_{UY|S=1} &= \frac{p(Y=1\mid A=1,U=1,S=1)}{p(Y=1\mid A=1,U=0,S=1)} \\ &= \frac{1-1/x}{1/\mathrm{BF}_1^*-1/x} \\ &= \frac{1-1/\mathrm{RR}_{AU|1}}{1/\mathrm{BF}_1^*-1/\mathrm{RR}_{AU|1}} \\ &= \frac{\mathrm{BF}_1^*(\mathrm{RR}_{AU|1}-1)}{\mathrm{RR}_{AU|1}-\mathrm{BF}_1^*}. \end{aligned}$$

We now have that

$$
\begin{aligned}
\mathrm{BF}_1 &= \frac{\mathrm{RR}_{AU|1} \times \mathrm{RR}_{UY|S=1}}{\mathrm{RR}_{AU|1} + \mathrm{RR}_{UY|S=1} - 1} \\
&= \frac{\mathrm{BF}_1^*(\mathrm{RR}_{AU|1} - 1)\mathrm{RR}_{AU|1}}{\mathrm{RR}_{AU|1}(\mathrm{RR}_{AU|1} - \mathrm{BF}_1^*) + \mathrm{BF}_1^*(\mathrm{RR}_{AU|1} - 1) - (\mathrm{RR}_{AU|1} - \mathrm{BF}_1^*)} \\
&= \mathrm{BF}_1^*.
\end{aligned}
$$

Finally, we have that

$$
\begin{aligned}
&E\{p(Y = 1 \mid A = 1, S = 1, U) \mid A = 0, S = 1\} \\
&\quad = p(Y = 1 \mid A = 1, S = 1, U = 1)p(U = 1 \mid A = 0, S = 1) \\
&\quad + p(Y = 1 \mid A = 1, S = 1, U = 0)p(U = 0 \mid A = 0, S = 1) \\
&\quad = p^*(Y = 1 \mid A = 1, S = 1) \times 1/x \\
&\quad + p^*(Y = 1 \mid A = 1, S = 1)\frac{1/\mathrm{BF}_1^* - 1/x}{1 - 1/x} \times (1 - 1/x) \\
&\quad = p^*(Y = 1 \mid A = 1, S = 1)/\mathrm{BF}_1^* \\
&\quad = p(Y = 1 \mid A = 1, S = 1)/\mathrm{BF}_1
\end{aligned}
$$

and

$$
\begin{aligned}
&E\{p(Y = 1 \mid A = 0, S = 1, U) \mid A = 1, S = 1\} \\
&\quad = p(Y = 1 \mid A = 0, S = 1, U = 1)p(U = 1 \mid A = 1, S = 1) \\
&\quad + p(Y = 1 \mid A = 0, S = 1, U = 0)p(U = 0 \mid A = 1, S = 1) \\
&\quad = 1 \times 1 + \frac{p^*(Y = 1 \mid A = 1, S = 1) - 1/x}{1 - 1/x} \times 0 \\
&\quad = 1,
\end{aligned}
$$

so that

$$
\begin{aligned}
&p(Y^1 = 1 \mid S = 1) = E\{p(Y = 1 \mid A = 1, S = 1, U)\} \\
&\quad = p(Y = 1 \mid A = 1, S = 1)p(A = 1 \mid S = 1) \\
&\quad + E\{p(Y = 1 \mid A = 1, S = 1, U) \mid A = 0, S = 1\}p(A = 0 \mid S = 1) \\
&\quad = p(Y = 1 \mid A = 1, S = 1)\{p(A = 1 \mid S = 1) + p(A = 0 \mid S = 1)/\mathrm{BF}_1\} \\
&\quad = l_1'
\end{aligned}
$$

and

$$
\begin{aligned}
&p(Y^0 = 1 \mid S = 1) = E\{p(Y = 1 \mid A = 0, S = 1, U)\} \\
&\quad = p(Y = 1 \mid A = 0, S = 1)p(A = 0 \mid S = 1) \\
&\quad + E\{p(Y = 1 \mid A = 0, S = 1, U) \mid A = 1, S = 1\}p(A = 1 \mid S = 1) \\
&\quad = p(Y = 1 \mid A = 0, S = 1)\{p(A = 0 \mid S = 1) + p(A = 1 \mid S = 1)/p(Y = 1 \mid A = 0, S = 1)\} \\
&\quad = u_0'.
\end{aligned}
$$

Table 2. Results for CRR_T with $\sigma = 3$. p_L and p_U are the proportions that SV's lower and upper bounds are equal to the sharp lower and upper bounds. Δ_L^{sharp}, Δ_U^{sharp}, Δ_L^{SV} and Δ_U^{SV} are the mean distance between $\log \mathrm{CRR}_T$ and the logarithm of bounds. CRR_T is the logarithm of the causal estimand.

$p(U=1)$	$p(A=1)$	$p(Y=1)$	$p(S=1)$	p_L	p_U	Δ_L^{sharp}	Δ_U^{sharp}	Δ_L^{SV}	Δ_U^{SV}	CRR_T
0.20	0.05	0.05	0.50	0	0	0.67	0.67	1.68	1.54	0.05
0.20	0.05	0.05	0.80	0	0	0.35	0.37	1.59	1.47	−0.10
0.20	0.05	0.20	0.50	0	0	0.51	0.50	1.41	1.32	−0.30
0.20	0.05	0.20	0.80	0	0	0.28	0.29	1.40	1.38	−0.44
0.20	0.20	0.05	0.50	0	0	0.64	0.70	1.60	1.56	0.12
0.20	0.20	0.05	0.80	0	0	0.34	0.37	1.74	1.63	0.02
0.20	0.20	0.20	0.50	0	0	0.53	0.53	1.40	1.34	−0.22
0.20	0.20	0.20	0.80	0	0	0.27	0.29	1.45	1.39	−0.25
0.50	0.05	0.05	0.50	0	0	0.67	0.70	1.85	1.74	−0.09
0.50	0.05	0.05	0.80	0	0	0.39	0.36	1.76	1.68	−0.01
0.50	0.05	0.20	0.50	0	0	0.56	0.54	1.59	1.45	−0.57
0.50	0.05	0.20	0.80	0	0	0.29	0.28	1.49	1.44	−0.45
0.50	0.20	0.05	0.50	0	0	0.68	0.66	1.84	1.65	0.44
0.50	0.20	0.05	0.80	0	0	0.35	0.34	1.93	1.69	0.33
0.50	0.20	0.20	0.50	0	0	0.58	0.53	1.66	1.50	−0.31
0.50	0.20	0.20	0.80	0	0	0.31	0.28	1.52	1.50	−0.29

K Additional numerical example with larger standard deviation

Table 3. Results for CRR_T with $\sigma = 3$. p_L and p_U are the proportions that SV's lower and upper bounds are equal to the alternative lower and upper bounds. Δ_L^{alt}, Δ_U^{alt}, Δ_L^{SV} and Δ_U^{SV} are the mean distance between $\log \mathrm{CRR}_T$ and the logarithm of bounds. CRR_T is the logarithm of the causal estimand.

$p(U=1)$	$p(A=1)$	$p(Y=1)$	$p(S=1)$	p_L	p_U	Δ_L^{alt}	Δ_U^{alt}	Δ_L^{SV}	Δ_U^{SV}	CRR_T
0.20	0.05	0.05	0.50	0.98	0.90	1.67	1.42	1.68	1.54	0.05
0.20	0.05	0.05	0.80	0.99	0.93	1.59	1.40	1.59	1.47	−0.10
0.20	0.05	0.20	0.50	0.94	0.86	1.34	1.17	1.41	1.32	−0.30
0.20	0.05	0.20	0.80	0.95	0.90	1.37	1.27	1.40	1.38	−0.44
0.20	0.20	0.05	0.50	0.99	0.93	1.59	1.46	1.60	1.56	0.12
0.20	0.20	0.05	0.80	1	0.96	1.74	1.59	1.74	1.63	0.02
0.20	0.20	0.20	0.50	0.94	0.88	1.33	1.20	1.40	1.34	−0.22
0.20	0.20	0.20	0.80	0.95	0.91	1.41	1.29	1.45	1.39	−0.25
0.50	0.05	0.05	0.50	0.99	0.90	1.84	1.63	1.85	1.74	−0.09
0.50	0.05	0.05	0.80	1	0.93	1.76	1.62	1.76	1.68	−0.01
0.50	0.05	0.20	0.50	0.91	0.86	1.54	1.32	1.59	1.45	−0.57
0.50	0.05	0.20	0.80	1	0.88	1.49	1.35	1.49	1.44	−0.45
0.50	0.20	0.05	0.50	0.98	0.94	1.83	1.57	1.84	1.65	0.44
0.50	0.20	0.05	0.80	1	0.95	1.93	1.65	1.93	1.69	0.33
0.50	0.20	0.20	0.50	0.92	0.85	1.60	1.35	1.66	1.50	−0.31
0.50	0.20	0.20	0.80	1	0.89	1.52	1.41	1.52	1.50	−0.29

Table 4. Results for CRD_T with $\sigma = 3$. p_L and p_U are the proportions that SV's lower and upper bounds are equal to the sharp lower and upper bounds. Δ_L^{sharp}, Δ_U^{sharp}, Δ_L^{SV} and Δ_U^{SV} are the mean distance between CRD_T and the bounds. CRD_T is the causal estimand.

$p(U=1)$	$p(A=1)$	$p(Y=1)$	$p(S=1)$	p_L	p_U	Δ_L^{sharp}	Δ_U^{sharp}	Δ_L^{SV}	Δ_U^{SV}	CRD_T
0.20	0.05	0.05	0.50	0	0	0.05	0.09	15.18	4.93	0.13
0.20	0.05	0.05	0.80	0	0	0.03	0.05	10.33	4.20	0.11
0.20	0.05	0.20	0.50	0	0	0.11	0.11	12.69	5.23	0.16
0.20	0.05	0.20	0.80	0	0	0.05	0.07	18.22	5.08	0.12
0.20	0.20	0.05	0.50	0	0	0.04	0.09	8.77	4.47	0.05
0.20	0.20	0.05	0.80	0	0	0.02	0.04	12.03	4.95	0.05
0.20	0.20	0.20	0.50	0	0	0.10	0.12	6.92	4.28	0.12
0.20	0.20	0.20	0.80	0	0	0.05	0.06	12.89	7.61	0.11
0.50	0.05	0.05	0.50	0	0	0.06	0.08	11.44	4.42	0.13
0.50	0.05	0.05	0.80	0	0	0.03	0.05	14.86	5.78	0.13
0.50	0.05	0.20	0.50	0	0	0.11	0.11	6.34	3.62	0.12
0.50	0.05	0.20	0.80	0	0	0.05	0.08	28.60	5.69	0.14
0.50	0.20	0.05	0.50	0	0	0.04	0.07	8.21	3.24	0.06
0.50	0.20	0.05	0.80	0	0	0.02	0.05	21.33	6.94	0.06
0.50	0.20	0.20	0.50	0	0	0.10	0.11	7.61	3.83	0.11
0.50	0.20	0.20	0.80	0	0	0.05	0.07	10.35	5.62	0.12

Table 5. Results for CRD_T with $\sigma = 3$. p_L and p_U are the proportions that SV's lower and upper bounds are equal to the alternative lower and upper bounds. Δ_L^{alt}, Δ_U^{alt}, Δ_L^{SV} and Δ_U^{SV} are the mean distance between CRD_T and the bounds. CRD_T is the causal estimand.

$p(U=1)$	$p(A=1)$	$p(Y=1)$	$p(S=1)$	p_L	p_U	Δ_L^{alt}	Δ_U^{alt}	Δ_L^{SV}	Δ_U^{SV}	CRD_T
0.20	0.05	0.05	0.50	0	0	0.13	0.17	15.18	4.93	0.13
0.20	0.05	0.05	0.80	0	0	0.12	0.15	10.33	4.20	0.11
0.20	0.05	0.20	0.50	0	0	0.26	0.23	12.69	5.23	0.16
0.20	0.05	0.20	0.80	0	0	0.24	0.23	18.22	5.08	0.12
0.20	0.20	0.05	0.50	0	0	0.10	0.15	8.77	4.47	0.05
0.20	0.20	0.05	0.80	0	0	0.09	0.14	12.03	4.95	0.05
0.20	0.20	0.20	0.50	0	0	0.24	0.23	6.92	4.28	0.12
0.20	0.20	0.20	0.80	0	0	0.24	0.22	12.89	7.61	0.11
0.50	0.05	0.05	0.50	0	0	0.15	0.17	11.44	4.42	0.13
0.50	0.05	0.05	0.80	0	0	0.12	0.16	14.86	5.78	0.13
0.50	0.05	0.20	0.50	0	0	0.29	0.25	6.34	3.62	0.12
0.50	0.05	0.20	0.80	0	0	0.22	0.25	28.60	5.69	0.14
0.50	0.20	0.05	0.50	0	0	0.12	0.15	8.21	3.24	0.06
0.50	0.20	0.05	0.80	0	0	0.08	0.13	21.33	6.94	0.06
0.50	0.20	0.20	0.50	0	0	0.28	0.26	7.61	3.83	0.11
0.50	0.20	0.20	0.80	0	0	0.22	0.25	10.35	5.62	0.12

Table 6. Results for CRR_S with $\sigma = 3$. p_L and p_U are the proportions that SV's lower and upper bounds are equal to the sharp lower and upper bounds. Δ_L^{sharp}, Δ_U^{sharp}, Δ_L^{SV} and Δ_U^{SV} are the mean distance between $\log \mathrm{CRR}_S$ and the logarithm of bounds. CRR_S is the logarithm of the causal estimand.

$p(U=1)$	$p(A=1)$	$p(Y=1)$	$p(S=1)$	p_L	p_U	Δ_L^{sharp}	Δ_U^{sharp}	Δ_L^{SV}	Δ_U^{SV}	CRR_S
0.20	0.05	0.05	0.50	1	0.90	0.46	0.56	0.46	0.66	0.04
0.20	0.05	0.05	0.80	1	0.94	0.31	0.42	0.31	0.48	−0.10
0.20	0.05	0.20	0.50	0.98	0.78	0.38	0.42	0.39	0.59	−0.31
0.20	0.05	0.20	0.80	1	0.85	0.27	0.34	0.27	0.46	−0.45
0.20	0.20	0.05	0.50	0.99	0.94	0.48	0.61	0.48	0.67	0.11
0.20	0.20	0.05	0.80	1	0.97	0.34	0.45	0.34	0.48	0.01
0.20	0.20	0.20	0.50	0.96	0.84	0.42	0.46	0.45	0.59	−0.23
0.20	0.20	0.20	0.80	0.99	0.89	0.29	0.34	0.30	0.43	−0.25
0.50	0.05	0.05	0.50	1	0.91	0.48	0.64	0.48	0.72	−0.12
0.50	0.05	0.05	0.80	1	0.93	0.34	0.40	0.34	0.47	−0.01
0.50	0.05	0.20	0.50	0.97	0.80	0.45	0.47	0.46	0.62	−0.58
0.50	0.05	0.20	0.80	1	0.83	0.26	0.29	0.26	0.41	−0.45
0.50	0.20	0.05	0.50	0.99	0.95	0.56	0.62	0.56	0.66	0.44
0.50	0.20	0.05	0.80	1	0.97	0.32	0.38	0.32	0.41	0.33
0.50	0.20	0.20	0.50	0.96	0.84	0.50	0.48	0.52	0.61	−0.31
0.50	0.20	0.20	0.80	1	0.87	0.31	0.32	0.31	0.44	−0.29

Table 7. Results for CRD_S with $\sigma = 3$. p_L and p_U are the proportions that SV's lower and upper bounds are equal to the sharp lower and upper bounds. Δ_L^{sharp}, Δ_U^{sharp}, Δ_L^{SV} and Δ_U^{SV} are the mean distance between CRD_S and the bounds. CRD_S is the causal estimand.

$p(U=1)$	$p(A=1)$	$p(Y=1)$	$p(S=1)$	p_L	p_U	Δ_L^{sharp}	Δ_U^{sharp}	Δ_L^{SV}	Δ_U^{SV}	CRD_S
0.20	0.05	0.05	0.50	0	0	0.04	0.11	0.07	0.68	0.13
0.20	0.05	0.05	0.80	0	0	0.03	0.09	0.04	0.42	0.11
0.20	0.05	0.20	0.50	0	0	0.08	0.12	0.20	0.78	0.16
0.20	0.05	0.20	0.80	0	0	0.05	0.10	0.09	0.69	0.12
0.20	0.20	0.05	0.50	0	0	0.03	0.09	0.06	0.43	0.05
0.20	0.20	0.05	0.80	0	0	0.02	0.06	0.03	0.20	0.05
0.20	0.20	0.20	0.50	0	0	0.08	0.13	0.23	0.78	0.12
0.20	0.20	0.20	0.80	0	0	0.06	0.09	0.12	0.68	0.11
0.50	0.05	0.05	0.50	0	0	0.05	0.11	0.07	0.47	0.13
0.50	0.05	0.05	0.80	0	0	0.04	0.09	0.04	0.42	0.13
0.50	0.05	0.20	0.50	0	0	0.09	0.13	0.18	0.76	0.12
0.50	0.05	0.20	0.80	0	0	0.05	0.10	0.08	0.50	0.14
0.50	0.20	0.05	0.50	0	0	0.03	0.08	0.06	0.20	0.06
0.50	0.20	0.05	0.80	0	0	0.02	0.06	0.03	0.29	0.06
0.50	0.20	0.20	0.50	0	0	0.09	0.13	0.21	0.74	0.11
0.50	0.20	0.20	0.80	0	0	0.06	0.10	0.08	1.02	0.12

L R code for the empirical example

```
library(haven)
library(dplyr)
library(ggplot2)

### LOAD AND COMBINE THE DIETARY DATASETS ###
diet1 = read_xpt("C:/.../DR1IFF.XPT")
diet2 = read_xpt("C:/.../DR1IFF_B.XPT")
diet3 = read_xpt("C:/.../DR1IFF_C.XPT")
diet4 = read_xpt("C:/.../DR1IFF_D.XPT")
diet5 = read_xpt("C:/.../DR1IFF_E.XPT")
diet6 = read_xpt("C:/.../DR1IFF_F.XPT")
diet7 = read_xpt("C:/.../DR1IFF_G.XPT")
diet8 = read_xpt("C:/.../DR1IFF_H.XPT")
diet9 = read_xpt("C:/.../DR1IFF_I.XPT")
diet10 = read_xpt("C:/.../DR1IFF_J.XPT")

diet1 = subset(diet1, select = c(SEQN, DRD030, DRDDRSTS))
diet2 = subset(diet2, select = c(SEQN, DRD030Z, DRDDRSTZ))
diet3 = subset(diet3, select = c(SEQN, DR1_030Z, DR1DRSTZ))
diet4 = subset(diet4, select = c(SEQN, DR1_030Z, DR1DRSTZ))
diet5 = subset(diet5, select = c(SEQN, DR1_030Z, DR1DRSTZ))
diet6 = subset(diet6, select = c(SEQN, DR1_030Z, DR1DRSTZ))
diet7 = subset(diet7, select = c(SEQN, DR1_030Z, DR1DRSTZ))
diet8 = subset(diet8, select = c(SEQN, DR1_030Z, DR1DRSTZ))
diet9 = subset(diet9, select = c(SEQN, DR1_030Z, DR1DRSTZ))
diet10 = subset(diet10, select = c(SEQN, DR1_030Z, DR1DRSTZ))

diet1$breakfastDummy = ifelse((diet1$DRD030==1|diet1$DRD030==9), 1, 0)
diet2$breakfastDummy = ifelse((diet2$DRD030Z==1|diet2$DRD030Z==10), 1, 0)
diet3$breakfastDummy = ifelse((diet3$DR1_030Z==1|diet3$DR1_030Z==10), 1, 0)
diet4$breakfastDummy = ifelse((diet4$DR1_030Z==1|diet4$DR1_030Z==10), 1, 0)
diet5$breakfastDummy = ifelse((diet5$DR1_030Z==1|diet5$DR1_030Z==10), 1, 0)
diet6$breakfastDummy = ifelse((diet6$DR1_030Z==1|diet6$DR1_030Z==10), 1, 0)
diet7$breakfastDummy = ifelse((diet7$DR1_030Z==1|diet7$DR1_030Z==10), 1, 0)
diet8$breakfastDummy = ifelse((diet8$DR1_030Z==1|diet8$DR1_030Z==10), 1, 0)
diet9$breakfastDummy = ifelse((diet9$DR1_030Z==1|diet9$DR1_030Z==10), 1, 0)
diet10$breakfastDummy = ifelse((diet10$DR1_030Z==1|diet10$DR1_030Z==10), 1, 0)

colnames(diet1) = c("SEQN", "DR1_030Z", "DR1DRSTZ")
colnames(diet2) = c("SEQN", "DR1_030Z", "DR1DRSTZ")

diet = rbind(diet1, diet2)
diet = rbind(diet, diet3)
diet = rbind(diet, diet4)
diet = rbind(diet, diet5)
diet = rbind(diet, diet6)
diet = rbind(diet, diet7)
diet = rbind(diet, diet8)
diet = rbind(diet, diet9)
diet = rbind(diet, diet10)

# Construct the breakfast-variable.
counter = 0
for (id in diet$SEQN) # Takes ~40 minutes per dataset.
{
  counter = counter + 1
  diet$breakfast[diet$SEQN==id] = ifelse(sum(diet$breakfastDummy[diet$SEQN==id])>0, 1, 0)
  print(counter)
}
```

```
diet = subset(diet, select = c(SEQN, breakfast, DR1DRSTZ))

# Remove duplicates.
diet = unique(diet)

# Save the dietary dataset.
save(diet, file = "diet.RData")

### LOAD AND COMBINE THE DEMOGRAPHIC DATASETS ###
demo1 = read_xpt("C:/.../DEMO.XPT")
demo2 = read_xpt("C:/.../DEMO_B.XPT")
demo3 = read_xpt("C:/.../DEMO_c.XPT")
demo4 = read_xpt("C:/.../DEMO_D.XPT")
demo5 = read_xpt("C:/.../DEMO_E.XPT")
demo6 = read_xpt("C:/.../DEMO_F.XPT")
demo7 = read_xpt("C:/.../DEMO_G.XPT")
demo8 = read_xpt("C:/.../DEMO_H.XPT")
demo9 = read_xpt("C:/.../DEMO_I.XPT")
demo10 = read_xpt("C:/.../DEMO_J.XPT")

demo1 = subset(demo1, select = c(SEQN, DMDMARTL, RIDRETH1, RIDAGEYR, RIAGENDR))
demo2 = subset(demo2, select = c(SEQN, DMDMARTL, RIDRETH1, RIDAGEYR, RIAGENDR))
demo3 = subset(demo3, select = c(SEQN, DMDMARTL, RIDRETH1, RIDAGEYR, RIAGENDR))
demo4 = subset(demo4, select = c(SEQN, DMDMARTL, RIDRETH1, RIDAGEYR, RIAGENDR))
demo5 = subset(demo5, select = c(SEQN, DMDMARTL, RIDRETH1, RIDAGEYR, RIAGENDR))
demo6 = subset(demo6, select = c(SEQN, DMDMARTL, RIDRETH1, RIDAGEYR, RIAGENDR))
demo7 = subset(demo7, select = c(SEQN, DMDMARTL, RIDRETH1, RIDAGEYR, RIAGENDR))
demo8 = subset(demo8, select = c(SEQN, DMDMARTL, RIDRETH1, RIDAGEYR, RIAGENDR))
demo9 = subset(demo9, select = c(SEQN, DMDMARTL, RIDRETH1, RIDAGEYR, RIAGENDR))
demo10 = subset(demo10, select = c(SEQN, DMDMARTL, RIDRETH1, RIDAGEYR, RIAGENDR))

demo = rbind(demo1, demo2)
demo = rbind(demo, demo3)
demo = rbind(demo, demo4)
demo = rbind(demo, demo5)
demo = rbind(demo, demo6)
demo = rbind(demo, demo7)
demo = rbind(demo, demo8)
demo = rbind(demo, demo9)
demo = rbind(demo, demo10)

# Save the demographic dataset.
save(demo, file = "demo.RData")

### LOAD AND COMBINE THE ALCOHOL DATASETS ###
alc1 = read_xpt("C:/.../ALQ.XPT")
alc2 = read_xpt("C:/.../ALQ_B.XPT")
alc3 = read_xpt("C:/.../ALQ_C.XPT")
alc4 = read_xpt("C:/.../ALQ_D.XPT")
alc5 = read_xpt("C:/.../ALQ_E.XPT")
alc6 = read_xpt("C:/.../ALQ_F.XPT")
alc7 = read_xpt("C:/.../ALQ_G.XPT")
alc8 = read_xpt("C:/.../ALQ_H.XPT")
alc9 = read_xpt("C:/.../ALQ_I.XPT")
alc10 = read_xpt("C:/.../ALQ_J.XPT")

alc1 = subset(alc1, select = c(SEQN, ALQ100))
alc2 = subset(alc2, select = c(SEQN, ALD100))
alc3 = subset(alc3, select = c(SEQN, ALQ101))
alc4 = subset(alc4, select = c(SEQN, ALQ101))
alc5 = subset(alc5, select = c(SEQN, ALQ101))
```

```
alc6 = subset(alc6, select = c(SEQN, ALQ101))
alc7 = subset(alc7, select = c(SEQN, ALQ101))
alc8 = subset(alc8, select = c(SEQN, ALQ101))
alc9 = subset(alc9, select = c(SEQN, ALQ101))
alc10 = subset(alc10, select = c(SEQN, ALQ121))

alc10$ALQ101 = ifelse(alc10$ALQ121==1|alc10$ALQ121==2|alc10$ALQ121==3|alc10$ALQ121==4|
    alc10$ALQ121==5|alc10$ALQ121==6|alc10$ALQ121==7, 1, 0)
alc10 = subset(alc10, select = c(SEQN, ALQ101))

colnames(alc1) = c("SEQN", "ALQ101")
colnames(alc2) = c("SEQN", "ALQ101")

alc = rbind(alc1, alc2)
alc = rbind(alc, alc3)
alc = rbind(alc, alc4)
alc = rbind(alc, alc5)
alc = rbind(alc, alc6)
alc = rbind(alc, alc7)
alc = rbind(alc, alc8)
alc = rbind(alc, alc9)
alc = rbind(alc, alc10)

# Save the alcohol dataset.
save(alc, file = "alc.RData")

### LOAD AND COMBINE THE SMOKING DATASETS ###
smoke1 = read_xpt("C:/.../SMQ.XPT")
smoke2 = read_xpt("C:/.../SMQ_B.XPT")
smoke3 = read_xpt("C:/.../SMQ_C.XPT")
smoke4 = read_xpt("C:/.../SMQ_D.XPT")
smoke5 = read_xpt("C:/.../SMQ_E.XPT")
smoke6 = read_xpt("C:/.../SMQ_F.XPT")
smoke7 = read_xpt("C:/.../SMQ_G.XPT")
smoke8 = read_xpt("C:/.../SMQ_H.XPT")
smoke9 = read_xpt("C:/.../SMQ_I.XPT")
smoke10 = read_xpt("C:/.../SMQ_J.XPT")

smoke1 = subset(smoke1, select = c(SEQN, SMQ020, SMQ040))
smoke2 = subset(smoke2, select = c(SEQN, SMQ020, SMQ040))
smoke3 = subset(smoke3, select = c(SEQN, SMQ020, SMQ040))
smoke4 = subset(smoke4, select = c(SEQN, SMQ020, SMQ040))
smoke5 = subset(smoke5, select = c(SEQN, SMQ020, SMQ040))
smoke6 = subset(smoke6, select = c(SEQN, SMQ020, SMQ040))
smoke7 = subset(smoke7, select = c(SEQN, SMQ020, SMQ040))
smoke8 = subset(smoke8, select = c(SEQN, SMQ020, SMQ040))
smoke9 = subset(smoke9, select = c(SEQN, SMQ020, SMQ040))
smoke10 = subset(smoke10, select = c(SEQN, SMQ020, SMQ040))

smoke = rbind(smoke1, smoke2)
smoke = rbind(smoke, smoke3)
smoke = rbind(smoke, smoke4)
smoke = rbind(smoke, smoke5)
smoke = rbind(smoke, smoke6)
smoke = rbind(smoke, smoke7)
smoke = rbind(smoke, smoke8)
smoke = rbind(smoke, smoke9)
smoke = rbind(smoke, smoke10)

# Save the smoking dataset.
save(smoke, file = "smoke.RData")
```

```
### LOAD AND COMBINE THE EXERCISE DATASETS ###
exercise1 = read_xpt("C:/.../PAQ.XPT")
exercise2 = read_xpt("C:/.../PAQ_B.XPT")
exercise3 = read_xpt("C:/.../PAQ_C.XPT")
exercise4 = read_xpt("C:/.../PAQ_D.XPT")
exercise5 = read_xpt("C:/.../PAQ_E.XPT")
exercise6 = read_xpt("C:/.../PAQ_F.XPT")
exercise7 = read_xpt("C:/.../PAQ_G.XPT")
exercise8 = read_xpt("C:/.../PAQ_H.XPT")
exercise9 = read_xpt("C:/.../PAQ_I.XPT")
exercise10 = read_xpt("C:/.../PAQ_J.XPT")

exercise1 = subset(exercise1, select = c(SEQN, PAD200))
exercise2 = subset(exercise2, select = c(SEQN, PAD200))
exercise3 = subset(exercise3, select = c(SEQN, PAD200))
exercise4 = subset(exercise4, select = c(SEQN, PAD200))
exercise5 = subset(exercise5, select = c(SEQN, PAQ650))
exercise6 = subset(exercise6, select = c(SEQN, PAQ650))
exercise7 = subset(exercise7, select = c(SEQN, PAQ650))
exercise8 = subset(exercise8, select = c(SEQN, PAQ650))
exercise9 = subset(exercise9, select = c(SEQN, PAQ650))
exercise10 = subset(exercise10, select = c(SEQN, PAQ650))

colnames(exercise1) = c("SEQN", "PAQ650")
colnames(exercise2) = c("SEQN", "PAQ650")
colnames(exercise3) = c("SEQN", "PAQ650")
colnames(exercise4) = c("SEQN", "PAQ650")

exercise = rbind(exercise1, exercise2)
exercise = rbind(exercise, exercise3)
exercise = rbind(exercise, exercise4)
exercise = rbind(exercise, exercise5)
exercise = rbind(exercise, exercise6)
exercise = rbind(exercise, exercise7)
exercise = rbind(exercise, exercise8)
exercise = rbind(exercise, exercise9)
exercise = rbind(exercise, exercise10)

# Save the exercise dataset.
save(exercise, file = "exercise.RData")

### LOAD AND COMBINE THE BODY MEASURES DATASETS ###
body1 = read_xpt("C:/.../BMX.XPT")
body2 = read_xpt("C:/.../BMX_B.XPT")
body3 = read_xpt("C:/.../BMX_C.XPT")
body4 = read_xpt("C:/.../BMX_D.XPT")
body5 = read_xpt("C:/.../BMX_E.XPT")
body6 = read_xpt("C:/.../BMX_F.XPT")
body7 = read_xpt("C:/.../BMX_G.XPT")
body8 = read_xpt("C:/.../BMX_H.XPT")
body9 = read_xpt("C:/.../BMX_I.XPT")
body10 = read_xpt("C:/.../BMX_J.XPT")

body1 = subset(body1, select = c(SEQN, BMXBMI))
body2 = subset(body2, select = c(SEQN, BMXBMI))
body3 = subset(body3, select = c(SEQN, BMXBMI))
body4 = subset(body4, select = c(SEQN, BMXBMI))
body5 = subset(body5, select = c(SEQN, BMXBMI))
body6 = subset(body6, select = c(SEQN, BMXBMI))
body7 = subset(body7, select = c(SEQN, BMXBMI))
```

```
body8 = subset(body8, select = c(SEQN, BMXBMI))
body9 = subset(body9, select = c(SEQN, BMXBMI))
body10 = subset(body10, select = c(SEQN, BMXBMI))

body = rbind(body1, body2)
body = rbind(body, body3)
body = rbind(body, body4)
body = rbind(body, body5)
body = rbind(body, body6)
body = rbind(body, body7)
body = rbind(body, body8)
body = rbind(body, body9)
body = rbind(body, body10)

# Save the body measures dataset.
save(body, file = "body.RData")

### MERGE ALL THE DATASETS ###
total = merge(demo, diet, by="SEQN")
total = merge(total, alc, by="SEQN")
total = merge(total, smoke, by="SEQN")
total = merge(total, exercise, by="SEQN")
total = merge(total, body, by="SEQN")

# Save the dataset.
save(total, file = "total.RData")

### MODIFY THE DATASET ###
# Remove non-reliable reponders.
analys = total[which(total$DR1DRSTZ==1|total$DR1DRSTZ==2),]
# Only keep non-hispanic whites.
analys = analys[which(analys$RIDRETH1==3),]
# Only keep age group 30-39.
analys = analys[which(analys$RIDAGEYR>29&analys$RIDAGEYR<40),]
# Remove women.
analys = analys[which(analys$RIAGENDR==1),]
# Non-smokers.
analys = analys[which(analys$SMQ020==2|analys$SMQ040==3),]
# Non-exercisers.
analys = analys[which(analys$PAQ650==2),]
# Create a categorical BMI variable.
analys$BMI = ifelse(analys$BMXBMI>25, 1, 0)
# Create a selection variable.
analys$sel = ifelse(analys$DMDMARTL==1|analys$DMDMARTL==6, 1, 0)
# Remove NA on the selection.
analys = analys[!is.na(analys$sel),]
# Only keep married and living partners.
selection = analys[which(analys$sel==1),]
# Save the smaller dataset.
save(selection, file = "selection.RData")

### CALCULATE BOUNDS FOR THE TOTAL POPULATION ###
# Calculate the probabilities.
pA1.S1 = length(selection$breakfast[selection$breakfast==1]) / length(selection$breakfast)
pA0.S1 = length(selection$breakfast[selection$breakfast==0]) / length(selection$breakfast)
pY1.A1S1 = length(selection$BMI[selection$BMI==1 & selection$breakfast==1]) /
    length(selection$breakfast[selection$breakfast==1])
pY1.A0S1 = length(selection$BMI[selection$BMI==1 & selection$breakfast==0]) /
    length(selection$breakfast[selection$breakfast==0])
pS1.A1 = length(analys$sel[analys$sel==1 & analys$breakfast==1]) /
    length(analys$breakfast[analys$breakfast==1])
```

```
pS1.A0 = length(analys$sel[analys$sel==1 & analys$breakfast==0]) /
    length(analys$breakfast[analys$breakfast==0])
pS0.A1 = 1 - pS1.A1
pS0.A0 = 1 - pS1.A0

# Relative risk of overweight for breakfasteaters vs non-breakfasteaters.
RRobs = pY1.A1S1 / pY1.A0S1

# Function for calculating BF from RR-parameters.
BFfunc <- function(RRSU, RRUY)
{
  BF = (RRSU * RRUY) / (RRSU + RRUY - 1)
}

x = y = seq(1, 10, by = 0.005)
z = outer(x, y, FUN = "BFfunc")

# Contour plot of BF as a function of RR.
par(mar=c(5,6,4,1)+.1)
contour(x, y, z,
        xlab=expression(RR[SUas]),
        ylab=expression(RR[UYa]),
        cex.lab = 2.5, cex.axis = 2.5,
        labcex = 2.5,
        lwd = 1.7)
grid()

# Function for calculating the upper bound in the total population from BF.
upperboundtot <- function(BF10, BF01)
{
  a = min(BF10, 1/pY1.A1S1)
  u1 = pY1.A1S1 * (pS1.A1 + pS0.A1 * a)
  l0 = pY1.A0S1 * (pS1.A0 + pS0.A0/BF01)

  bound = u1 / l0
  return(bound)
}

x2 = y2 = seq(1, 3, by = 0.005)

# Calculating the bound.
outmat = matrix(nrow = length(x2), ncol = length(y2), NA)
for (iii in 1:length(x2))
{
  for (jjj in 1:length(y2))
  {
    outmat[iii,jjj] = upperboundtot(x2[iii], y2[jjj])
  }
}

# Contour plot of the upper bound as a function of BF.
par(mar=c(5,6,4,1)+.1)
contour(x2, y2, outmat,
        xlab=expression(BF[10]),
        ylab=expression(BF["01"]),
        cex.lab = 2.5, cex.axis = 2.5,
        labcex = 2.5,
        lwd = 1.7)
grid()

# Function for calculating the upper bound in the subpopulation from RR.
```

```
upperboundsub <- function(RRAU0, RRUY)
{
  BF0 = RRAU0 * RRUY / (RRAU0 + RRUY - 1)
  u1 = pY1.A1S1 * (pA1.S1 + pA0.S1 * min(BF0, 1/pY1.A1S1))
  l0 = pY1.A0S1 * (pA0.S1 + pA1.S1/BF0)

  bound = u1 / l0
}

x3 = y3 = seq(1, 5, by = 0.005)

# Calculating the bound.
outmat2 = matrix(nrow = length(x3), ncol = length(y3), NA)
for (iii in 1:length(x3))
{
  for (jjj in 1:length(y3))
  {
    outmat2[iii,jjj] = upperboundsub(x3[iii], y3[jjj])
  }
}

# Contour plot of the upper bound as a function of RR.
par(mar=c(5,6,4,1)+.1)
contour(x3, y3, outmat2,
        method = "flattest",
        nlevels = 5,
        xlab=expression(RR[AU0]),
        ylab=expression(RR[UYS1]),
        cex.lab = 2.5, cex.axis = 2.5,
        labcex = 2.5,
        lwd = 1.7)
grid()

# Nonparametric bootstrap confidence intervals.
# Number of subjects in different groups.
nA1 = length(analys$breakfast[analys$breakfast==1])
nA0 = length(analys$breakfast[analys$breakfast==0])
nS1 = length(analys$breakfast[analys$sel==1])
nA1S1 = length(analys$sel[analys$sel==1 & analys$breakfast==1])
nA0S1 = length(analys$sel[analys$sel==1 & analys$breakfast==0])

# Function for calculating the upper bound in the total population from the RR-parameters
# and the probabilities.
boundfun <- function(RRSU01, RRSU10, RRUY1, RRUY0, pY1A0S1, pY1A1S1, pS1A1, pS1A0)
{
  BF01 = RRSU01 * RRUY0 / (RRSU01 + RRUY0 - 1)
  BF10 = RRSU10 * RRUY1 / (RRSU10 + RRUY1 - 1)
  pS0A0 = 1 - pS1A0
  pS0A1 = 1 - pS1A1
  bound = (pY1A1S1 * (pS1A1 + pS0A1 * min(BF10, (1/pY1A1S1)))) / (pY1A0S1 * (pS1A0 + pS0A0 / BF01))
}

# Range for RR.
r = seq(1, 10, 0.1)

# Storing matrix.
bound = matrix(nrow=length(r), ncol=3)

# Number of bootstrap samples.
B = 1000
```

```
# Bootstrap probabilities.
pS1.A1.boot = rbinom(n=B,size=nA1, prob=pS1.A1)/nA1
pS1.A0.boot = rbinom(n=B,size=nA0, prob=pS1.A0)/nA0
pY1.A1S1.boot = rbinom(n=B,size=nA1S1, prob=pY1.A1S1)/nA1S1
pY1.A0S1.boot = rbinom(n=B,size=nA0S1, prob=pY1.A0S1)/nA0S1
RRobs.boot = pY1.A1S1.boot/pY1.A0S1.boot

# Calculating the 95% bootstrap confidence intervals.
for(i in 1:length(r))
{
  bound[i, 1] = boundfun(r[i], r[i], r[i], r[i], pY1.A0S1, pY1.A1S1, pS1.A1, pS1.A0)
  bound.boot = boundfun(r[i], r[i], r[i], r[i], pY1.A0S1.boot, pY1.A1S1.boot, pS1.A1.boot, pS1.A0.boot)
  qq = quantile(x=bound.boot, probs=c(0.025, 0.975))
  bound[i, 2] = qq[1]
  bound[i, 3] = qq[2]
}

# Plot the upper bound for the total population and 95% CI when all RR are the same.
plot(0, xlim=c(min(r), max(r)), ylim=c(min(bound), max(bound)),
     type="n", xlab=bquote(RR[SU01]*"="*RR[SU10]*"="*RR[UY1]*"="*RR[UY0]),
     ylab="Upper bound", cex.lab = 2.5, cex.axis = 2.5)
grid()
matlines(r, bound, lty=c("solid", "dashed", "dashed"), col="black", lwd=2)
legend(x="topleft", lty=c("solid", "dashed"), lwd = 2, legend=c("Bound", "95% CI"),
       bty="n", cex = 2.5)

# Function for calculating the upper bound in the spopulation from the RR-parameters and the probabilities.
boundSubfun <- function(RRAU0, RRUYS, pY1A0S1, pY1A1S1, pA1S1)
{
  BF0 = RRAU0 * RRUYS / (RRAU0 + RRUYS - 1)
  pA0S1 = 1 - pA1S1
  bound = (pY1A1S1 * (pA1S1 + pA0S1 * min(BF0, (1/pY1A1S1)))) / (pY1A0S1 * (pA0S1 + pA1S1 / BF0))
}

# Storing matrix.
boundSub = matrix(nrow=length(r), ncol=3)

# Bootstrap probabilities.
pA1.S1.boot = rbinom(n=B,size=nS1, prob=pA1.S1)/nS1
pY1.A1S1.boot = rbinom(n=B,size=nA1S1, prob=pY1.A1S1)/nA1S1
pY1.A0S1.boot = rbinom(n=B,size=nA0S1, prob=pY1.A0S1)/nA0S1
RRobs.boot = pY1.A1S1.boot/pY1.A0S1.boot

# Calculating the 95% bootstrap confidence intervals.
for(i in 1:length(r))
{
  boundSub[i, 1] = boundSubfun(r[i], r[i], pY1.A0S1, pY1.A1S1, pA1.S1)
  bound.boot = boundSubfun(r[i], r[i], pY1.A0S1.boot, pY1.A1S1.boot, pA1.S1.boot)
  qq = quantile(x=bound.boot, probs=c(0.025, 0.975))
  boundSub[i, 2] = qq[1]
  boundSub[i, 3] = qq[2]
}

# Plot the upper bound for the subpopulation and 95% CI when all RR are the same.
plot(0, xlim=c(min(r), max(r)), ylim=c(min(boundSub), max(boundSub)),
     type="n", xlab=bquote(RR[AU0]*"="*RR[UYS1]),
     ylab="Upper bound", cex.lab = 2.5, cex.axis = 2.5)
matlines(r, boundSub, lty=c("solid", "dashed", "dashed"), col="black", lwd=2)
legend(x="topleft", lty=c("solid", "dashed"), lwd = 2, legend=c("Bound", "95% CI"),
       bty="n", cex = 2.5)
grid()
```

References

Balke, A. and J. Pearl (1994). Probabilistic evaluation of counterfactual queries. In *Proceedings of the 12th Conference on Artificial Intelligence*, pp. 230–237.

Ding, P. and T. Vanderweele (2016). Sharp sensitivity bounds for mediation under unmeasured mediator-outcome confounding. *Biometrika 103*(2), 483–490.

Ding, P. and T. J. VanderWeele (2016). Sensitivity analysis without assumptions. *Epidemiology 27*(3), 368–377.

Smith, L. and T. VanderWeele (2019). Bounding bias due to selection. *Epidemiology 30*(4), 509–516.
